# Recent advances in combining photo- and N-heterocyclic carbene catalysis

**DOI:** 10.1039/d3sc03274d

**Published:** 2023-10-31

**Authors:** Xiaochen Wang, Senhui Wu, Rongxin Yang, Hongjian Song, Yuxiu Liu, Qingmin Wang

**Affiliations:** a State Key Laboratory of Elemento-Organic Chemistry, Research Institute of Elemento-Organic Chemistry, Frontiers Science Center for New Organic Matter, College of Chemistry, Nankai University Tianjin 300071 People's Republic of China wangqm@nankai.edu.cn

## Abstract

N-Heterocyclic carbenes (NHCs) are unique Lewis basic catalysts that mediate various organic transformations by means of polarity reversal. Although the scope of research on two-electron reactions mediated by NHC catalysts has been expanding, the types of these reactions are limited by the inability of NHCs to engage sp^3^-electrophiles. However, the revival of photocatalysis has accelerated the development of free-radical chemistry, and combining photoredox catalysis and NHC catalysis to achieve NHC-mediated radical reactions under mild conditions could overcome the above-mentioned limitation. This review summarizes recent advances in combining photoredox and NHC catalysis, focusing on elucidation and exploration of mechanisms, with the aim of identifying challenges and opportunities to develop more types of catalytic models.

## Abstract 2

This review summarizes recent advances in combining photo- and N-heterocyclic carbene catalysis, as well as provides an outlook on future opportunities and challenges.

## Graphical abstract

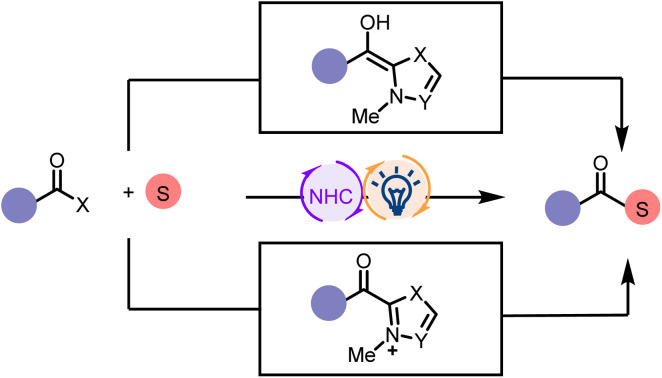

## 1. Introduction

**Scheme 1:**
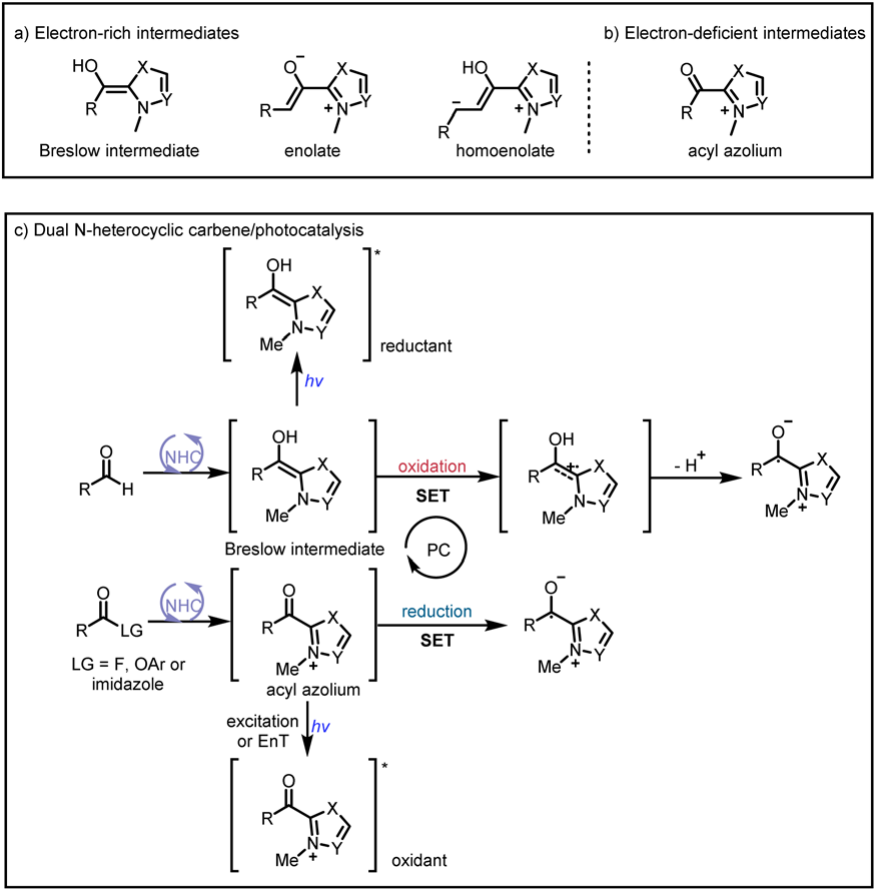
(a) Typical electron-rich NHC-bound intermediates. (b) Typical electron-deficient NHC-bound intermediates. (c) Main modes of NHC/photoredox dual catalysis.

Organic small-molecule catalysts can simulate the activity of enzymes and are inexpensive, readily available, mild, and less toxic than metal catalysts. In particular, N-heterocyclic carbenes (NHCs) have been successfully used as organocatalysts over the past few decades because of their versatility and unique structure and been a research hotspot in the field of catalysis.^1^ On the basis of their general properties and chemical applications, NHC-bound intermediates can be broadly divided into two types: electron-rich and electron-deficient (Scheme 1a and b, respectively).^1*e*^ The traditional mode of NHC catalysis typically involves direct participation of such intermediates in reactions to form chemical bonds through a two-electron process; however, the scope with respect to NHC-derived operators is restricted by their inability to engage sp^3^ electrophiles.^2^ Therefore, the discovery of new reaction modes for NHC catalysts, such as single-electron transfer (SET) radical reactions, will bring about new reaction modes to NHC catalysis and also rejuvenate its vitality and vigour.

Recent advances in single-electron reactions involving photoredox catalysis^3^ have enabled bond connections that were previously not possible by means of traditional methods, and these advances have been used for organic synthesis.^4^ The revival of photocatalysis research has accelerated the development of free-radical chemistry, and visible-light catalysis has been combined with NHC catalysis to achieve NHC-mediated radical reactions under mild conditions. In recent years, the cocatalysis model has been in a state of explosion, and there are some reviews in related fields.^5^ Although there have some big shoes to fill, our review focuses on the summary of the newly developed catalytic mechanism, especially in the last four years. At present, there are two main modes of NHC/photoredox dual catalysis: (1) single-electron oxidation of classical electron-rich NHC intermediates to generate ketyl radicals under photocatalytic conditions and (2) single-electron reduction of novel NHC-derived electron-deficient radical intermediates under photocatalytic conditions. Among them, the electron-rich or electron-deficient intermediates can be used as oxidizing or reducing agents after direct photoexcitation (Scheme 1c, top and bottom, respectively). And the generated ketyl radicals will engage in radical/radical cross-couplings; although not employing light activation directly, the pioneering work of Ohmiya and co-workers based on a NHC-derived ketyl radical using NHPI esters as oxidative radical precursors under thermal conditions will also be discussed.

This review is divided into two main sections. The first is dedicated to production of ketyl radicals by single-electron oxidation (*via* Breslow intermediates), and the second focuses on generation of ketyl radicals by single-electron reduction (*via* acyl azolium intermediates).

## 2. Ketyl radicals generated *via* Breslow intermediates

**Scheme 2:**
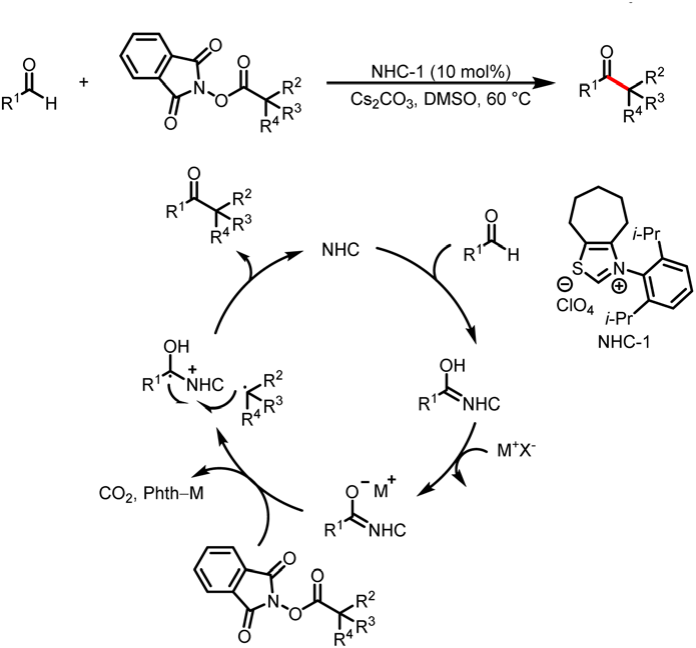
Decarboxylative alkylation of aldehydes.

Although not employing light activation directly, as pioneering work, in 2019, Ohmiya reported the first example of radical cross-coupling reactions based on a NHC-catalyzed decarboxylative coupling reaction between aryl aldehydes and tertiary or secondary alkyl carboxylic acid-derived redox-active esters to deliver aryl alkyl ketone (Scheme 2).^6^ The Breslow intermediate formed from the aldehyde and NHC reduces the NHPI ester to afford a ketyl radical and an alkyl radical, and the radical–radical coupling between them followed by the elimination of NHC will afford the desired ketone product.

### 2.1 Reactions requiring an external photocatalyst

#### 2.1.1 Aldehydes as substrates

**Scheme 3:**
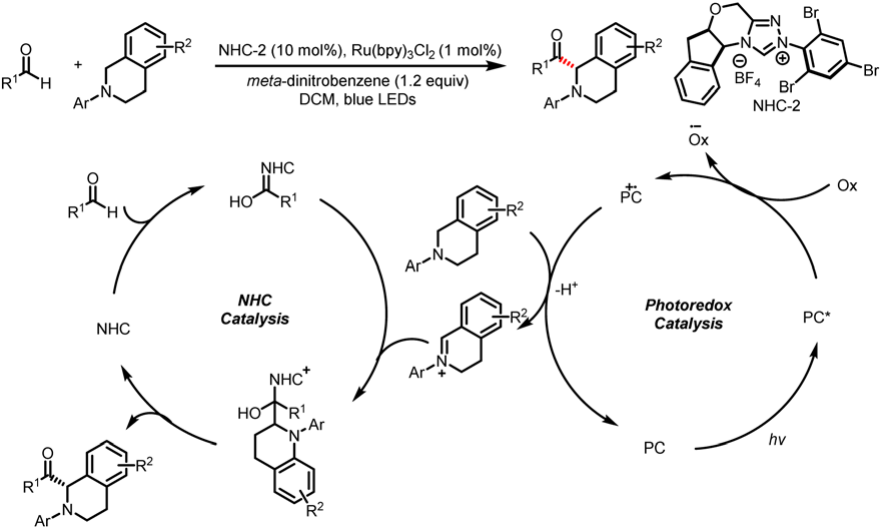
Asymmetric α-acylation of tertiary amines.

For the oxidation pathway, NHC addition into an aldehyde generates a Breslow intermediate, which can then reduce a radical acceptor to generate an NHC-derived ketyl radical species. In 2012, the Rovis group realized the first combination of NHC catalysis and photocatalysis, and this dual-catalysis mode enables the catalytic asymmetric α-acylation of cyclic tertiary amines to generate α-amino ketones (Scheme 3).^7^ Two chemically distinct activation modes eventually result in the formation of the desired C–C bond, with H_2_ as the only by-product. Mechanistic studies indicated that irradiation of the photocatalyst with blue light populates the excited state, which is oxidized by *meta*-dinitrobenzene; the oxidized photocatalyst is reduced by the tertiary amine, and subsequent hydrogen atom abstraction results in the formation of an iminium ion. The reaction of the NHC with the aldehyde generates a nucleophilic Breslow intermediate, which is intercepted by the iminium ion to forge a new C–C bond. The elimination of the NHC provides the α-amino ketone product and completes the catalytic cycle.

**Scheme 4:**
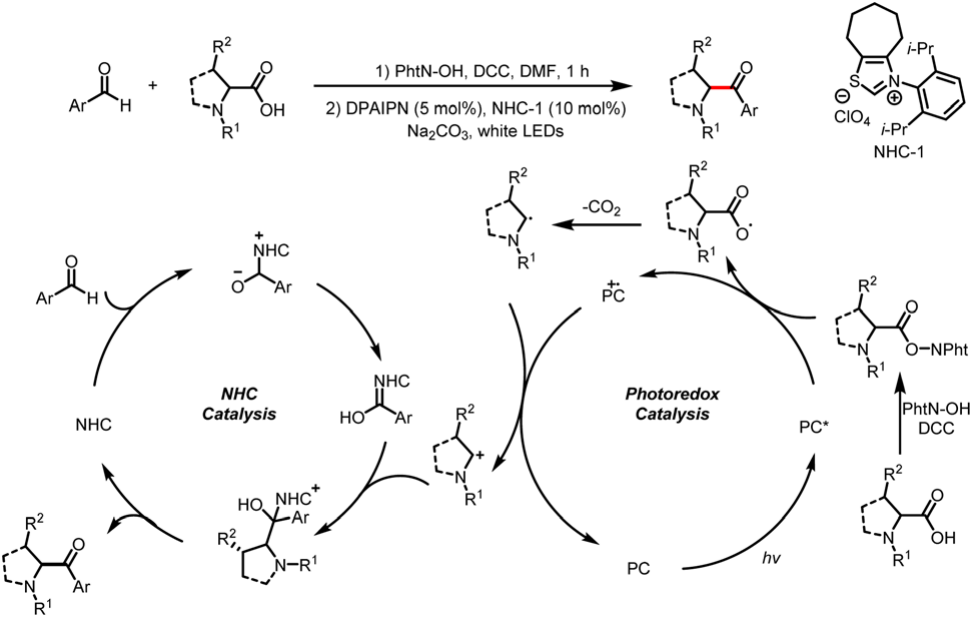
Decarboxylative carbonylation of α-amino acids.

The Du group described modification of α-amino acids and peptides with aldehydes by photoredox/NHC dual catalysis to access structurally diverse α-amino ketones (Scheme 4).^8^ Amino acids prepared in advance or generated *in situ* act as radical precursors. These reactions proceed by a mechanism similar to that described by Rovis *et al.*^7^

**Scheme 5:**
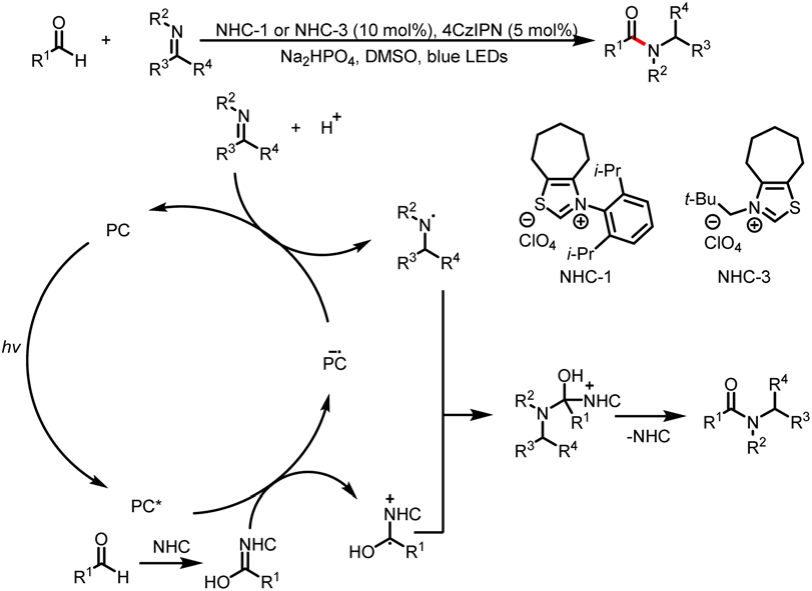
Amide synthesis from aldehydes and imines.

In 2020, Shu and a co-workers reported the direct synthesis of amides from aldehydes and imines under redox-neutral conditions at room temperature. The key to the success of this method is NHC/visible light dual catalysis, which enables photocatalytic reduction of imino esters to nitrogen-centered radicals, which react with radical cationic intermediates to form C–N bonds (Scheme 5).^9^

**Scheme 6:**
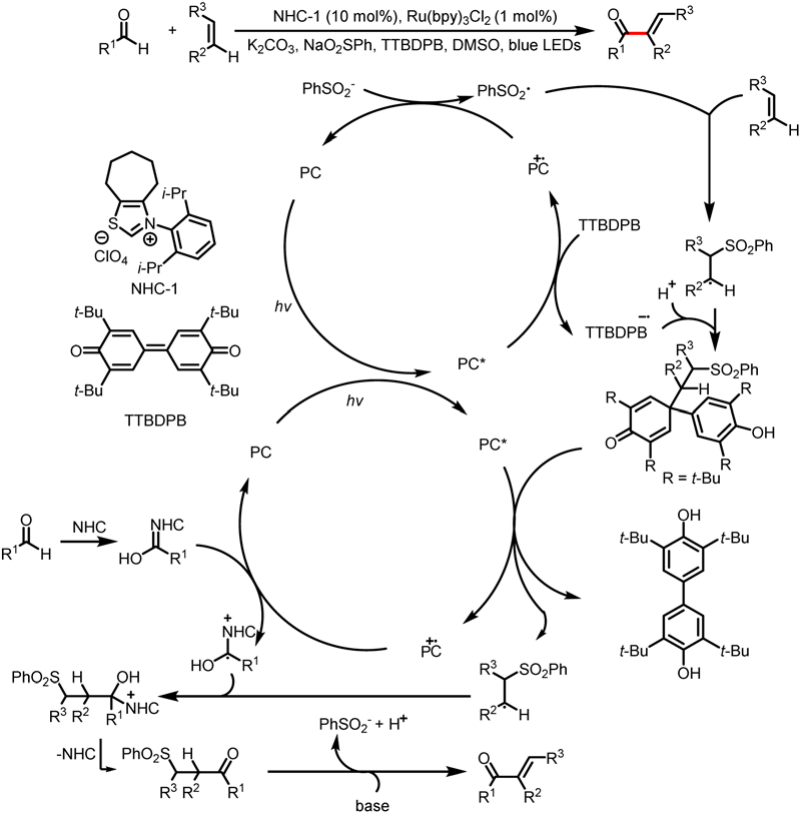
Direct coupling of aldehydes with alkenes.

Shortly thereafter, Shu's group reported intermolecular vinylation of aldehydes, which was achieved by two sequential C–H functionalization reactions involving alkenes as vinylating reagents (Scheme 6).^10^ The reaction conditions are compatible with aldehydes and olefins bearing a wide range of functional groups. It is worth mentioning that TTBDPB (3,3′,5,5′-tetra-*tert*-butyldiphenoquinone), which is used in these reactions, acts both as an oxidant and as a reservoir for radical intermediates.

**Scheme 7:**
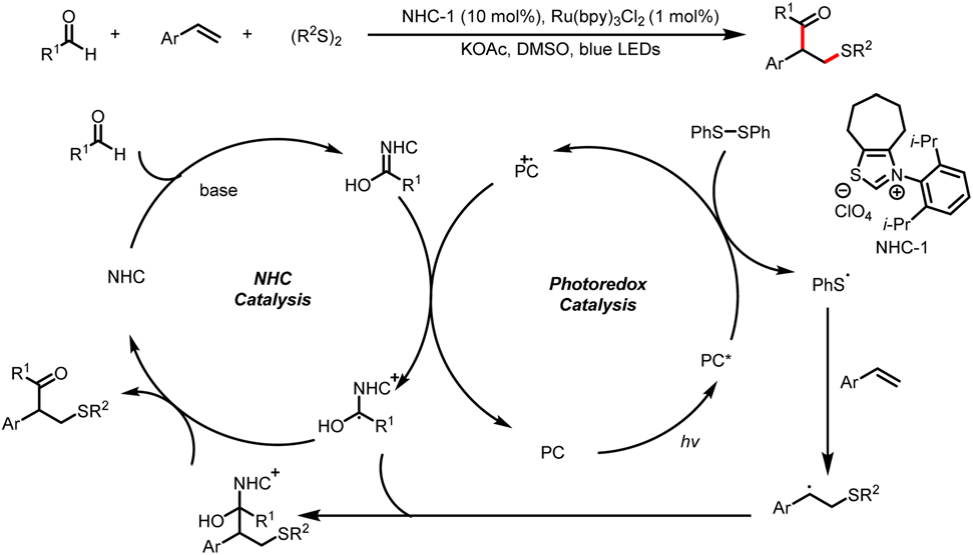
Synthesis of α-arylated-β-thiolated ketones.

Along with this two-component coupling process, Shu and co-workers also developed a related three-component protocol for straightforward access to β-thiolated ketones from aldehydes, styrenes, and disulfides (Scheme 7).^11^ First, a Breslow intermediate undergoes single-electron oxidation by the excited-state photocatalyst, and then reduction of a diaryl disulfide generates a sulfur radical. The addition of the sulfur radical to a styrene derivative forms a new carbon-centered radical intermediate, which undergoes radical cross-coupling with a ketyl radical to afford the α-arylated-β-thiolated ketone product. A more stable benzyl radical was obtained by the addition with styrene analogues.

**Scheme 8:**
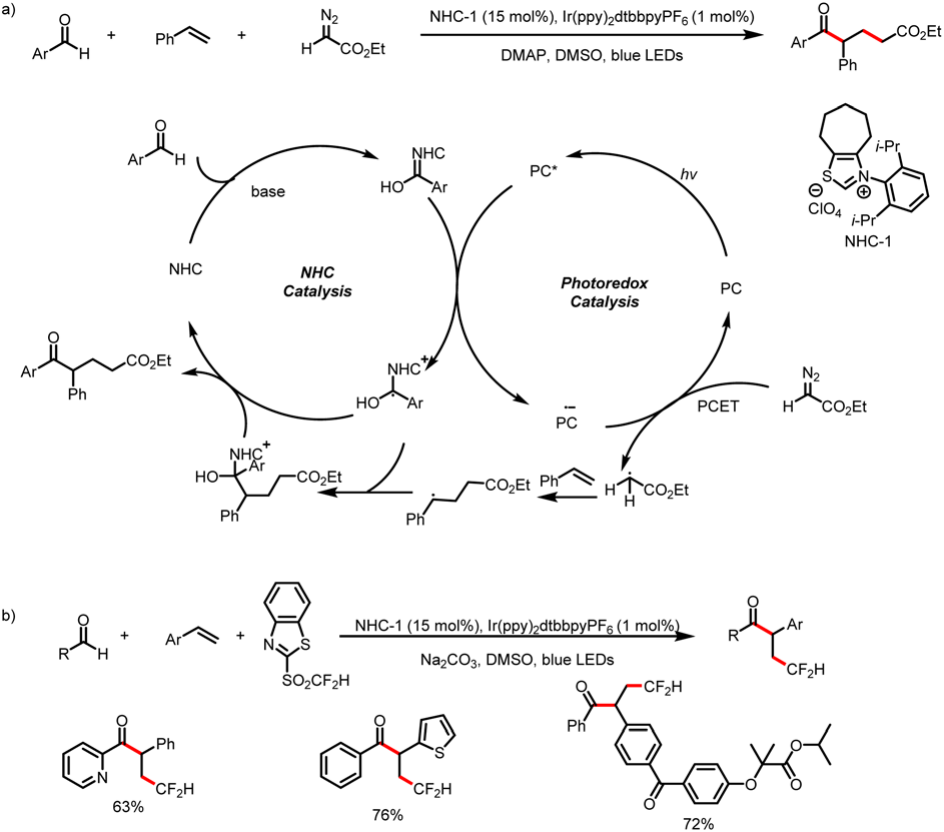
(a) Bisfunctionalization of unactivated olefins by aldehydes and a diazoester. (b) Bisfunctionalization of unactivated olefins by aldehydes and 2-((difluoromethyl)-sulfonyl)benzo[*d*]thiazole.

Meanwhile, the Wang group also developed a method for three-component bisfunctionalization of unactivated olefins by means of NHC/photoredox dual catalysis. Proton-coupled electron transfer (PCET) generates free radicals from a diazo ester, and the radicals add to olefins to generate new radicals that couple with ketyl radicals generated *via* photoredox-catalyzed oxidation of Breslow intermediates (Scheme 8a).^12^ In addition, acyldifluoromethylation of inert alkenes can be achieved *via* a similar mechanism when 2-((difluoromethyl)-sulfonyl)benzo[*d*]thiazole is used as a radical precursor (Scheme 8b).^13^

**Scheme 9:**
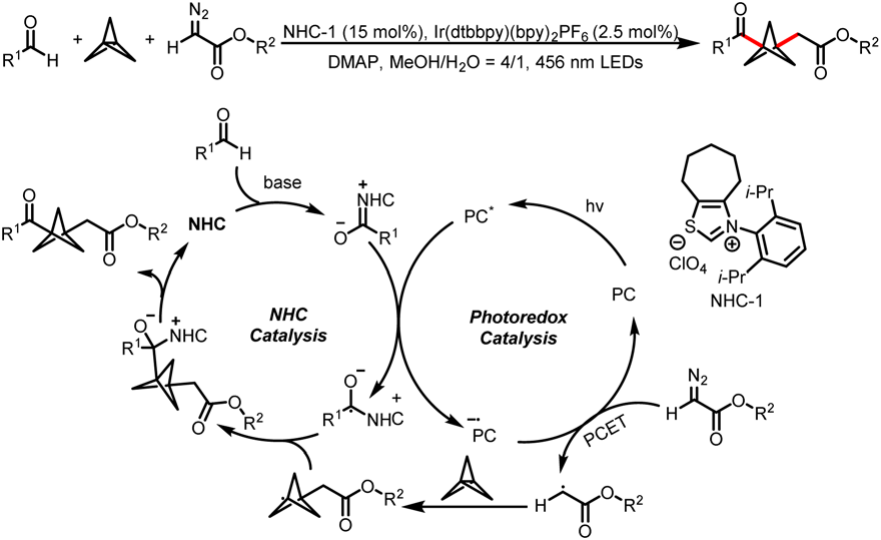
Synthesis of 1,3-disubstituted bicyclo[1.1.1]pentane ketones.

Recently, the Jiang and Yu group also developed a three-component reaction to access 1,3-disubstituted bicyclo[1.1.1]pentane (BCP) ketones (Scheme 9).^14^ The BCP scaffold is a bioisostere for the phenyl ring and could enhance the solubility and permeability of bioactive molecules. In their work, radicals derived from diazo esters perform an addition reaction onto [1.1.1] propellane to afford BCP radicals, which are then coupled with the ketyl radicals that are generated *via* oxidation of the Breslow intermediates by the photoredox catalyst.

#### 2.1.2 Enals as substrates

**Scheme 10:**
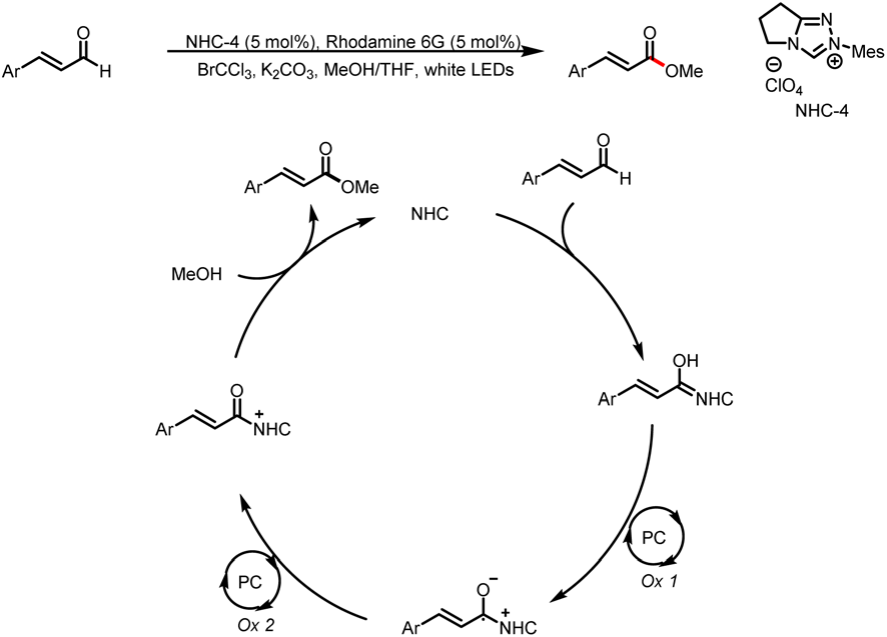
Chemoselective oxidative esterification of cinnamaldehydes.

In addition to aldehydes, enals have also been used as substrates to generate Breslow intermediates by a combination of NHC catalysis and photocatalysis. The distribution of radical features provided by conjugation in unsaturated Breslow intermediates can be further extended for remote functionalization. NHC addition into an enal generates a Breslow intermediate, which converted into its dienate form by the elimination of the pre-added leaving group. For example, in 2018, Miyabe's group reported chemoselective oxidative esterification of cinnamaldehydes by oxidation of Breslow intermediates with O_2_ (Scheme 10).^15^ This catalytic cycle is completed by continuous single-electron oxidation of the photocatalyst in the presence of methanol acting as a nucleophile.

**Scheme 11:**
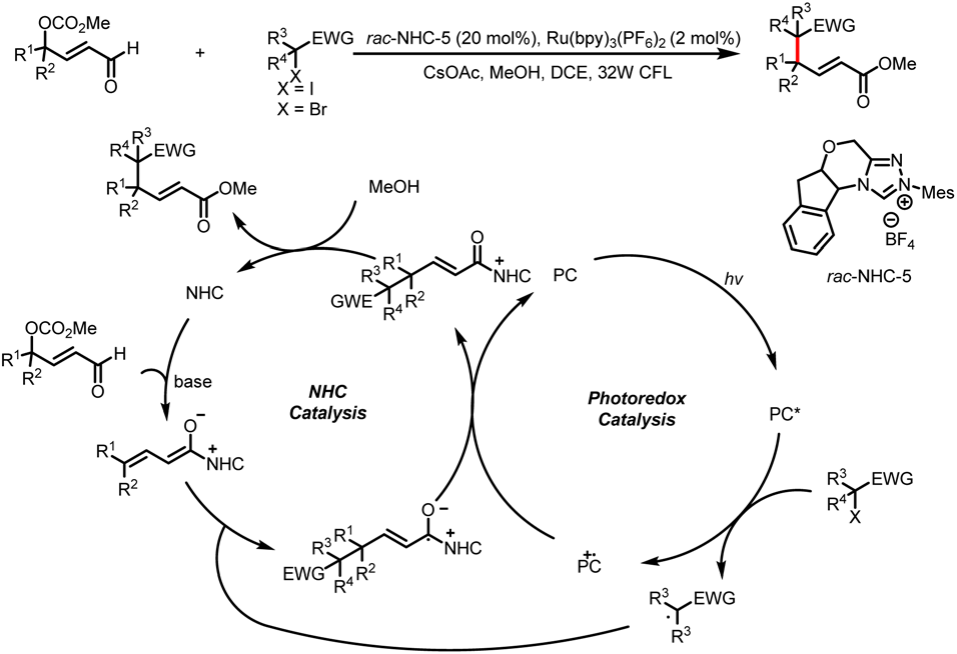
γ- and ε-alkylation of enals.

In 2019, the Ye group reported the use of γ-oxidized enals as substrates for the synthesis of γ-multisubstituted-α,β-unsaturated esters by intramolecular alkylation reactions with alkyl halides bearing an electron-withdrawing group (Scheme 11).^16^ An alkyl radical generated from the alkyl halide by photocatalysis reacts with a dienolate intermediate generated from the enal by NHC catalysis to give a homoenolate radical. The subsequent SET oxidation of the homoenolate radical mediated by the photocatalyst affords an α,β-unsaturated acyl azolium intermediate and completes the photocatalytic cycle. Trapping of the acyl azolium intermediate by methanol gives the final product and regenerates the NHC catalyst.

**Scheme 12:**
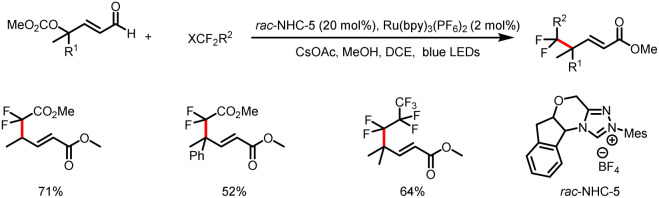
Synthesis of γ-difluoroalkyl-α,β-unsaturated esters.

The Ye group also developed a method for γ-difluoroalkylation of γ-preoxidized enals to afford γ-difluoroalkyl-α,β-unsaturated esters (Scheme 12).^17^ This method allows for efficient construction of C(sp^3^)–CF_2_R bonds at the γ position of carbonyl compounds bearing an all-carbon quaternary center.

**Scheme 13:**
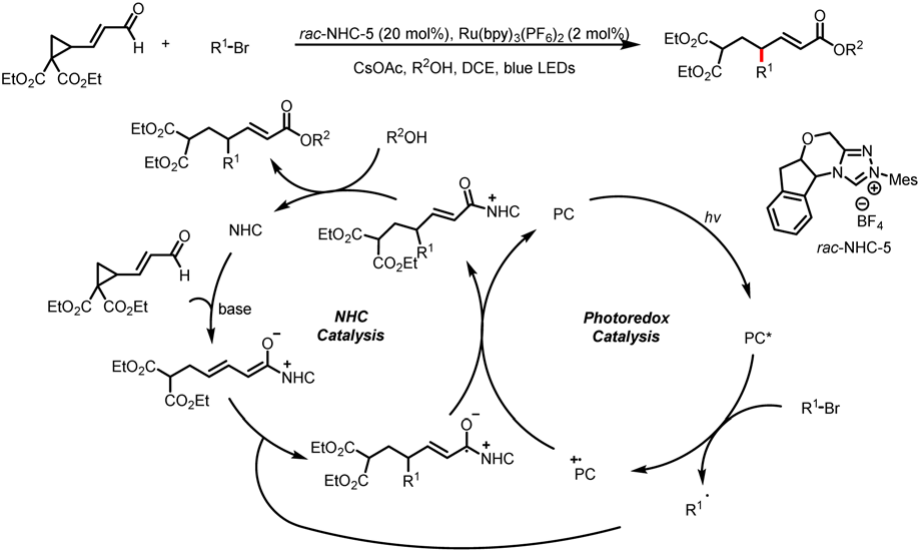
Synthesis of γ-alkyl-α,β-unsaturated esters.

Subsequently, Ye and a colleague expanded the substrate scope of the reaction to include cyclopropane enals. Initial ring-opening by C–C bond cleavage and subsequent γ-alkylation with a halogenated compound *via* a radical process afford γ-alkyl-α,β-unsaturated esters (Scheme 13).^18^ A variety of alkyl halides work well in the reaction, providing the desired γ-alkyl-α,β-unsaturated ester products in moderate to good yields.

**Scheme 14:**
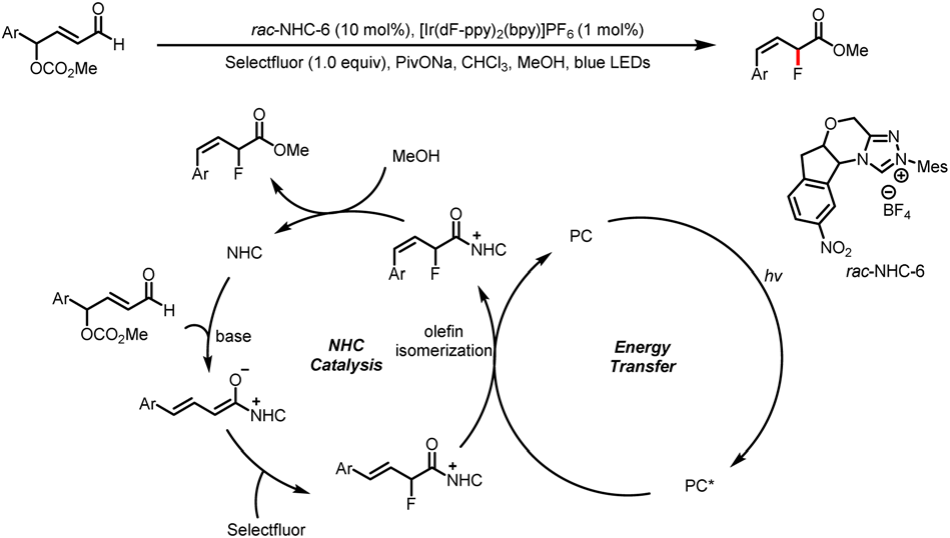
Synthesis of (*Z*)-allylic fluorides.

In 2021, Huang and Chen *et al.* reported a method for photoinduced energy-transfer reactions of NHC-bound intermediates to yield (*Z*)-allylic fluorides with good stereochemical control (Scheme 14).^19^ A homoenolate intermediate is generated by nucleophilic addition of an NHC to an enal substrate followed by a hydride shift. A dienolate formed by release of CO_2_ and methanol reacts with Selectfluor to give an acyl azolium intermediate. By energy transfer from the triplet excited state of the photocatalyst, the acyl azolium intermediate undergoes double bond isomerization to afford predominantly (*Z*)-acyl azolium, which reacts with MeOH to yield the product and regenerate the NHC catalyst.

**Scheme 15:**
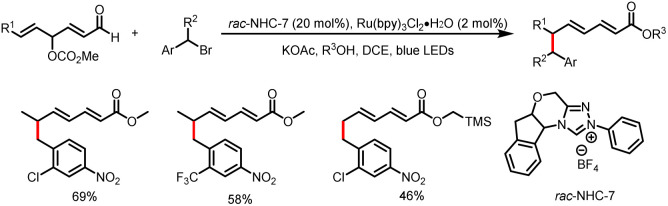
Synthesis of ε-benzyl-α,β-γ,δ-bisunsaturated esters.

Then Ye *et al.* extended the conjugate system further and realized regioselective ε-benzylation of γ-alkenyl-γ-oxidized enals to afford the corresponding ε-benzyl-α,β-γ,δ-bisunsaturated esters in moderate to good yields (Scheme 15).^20^

**Scheme 16:**
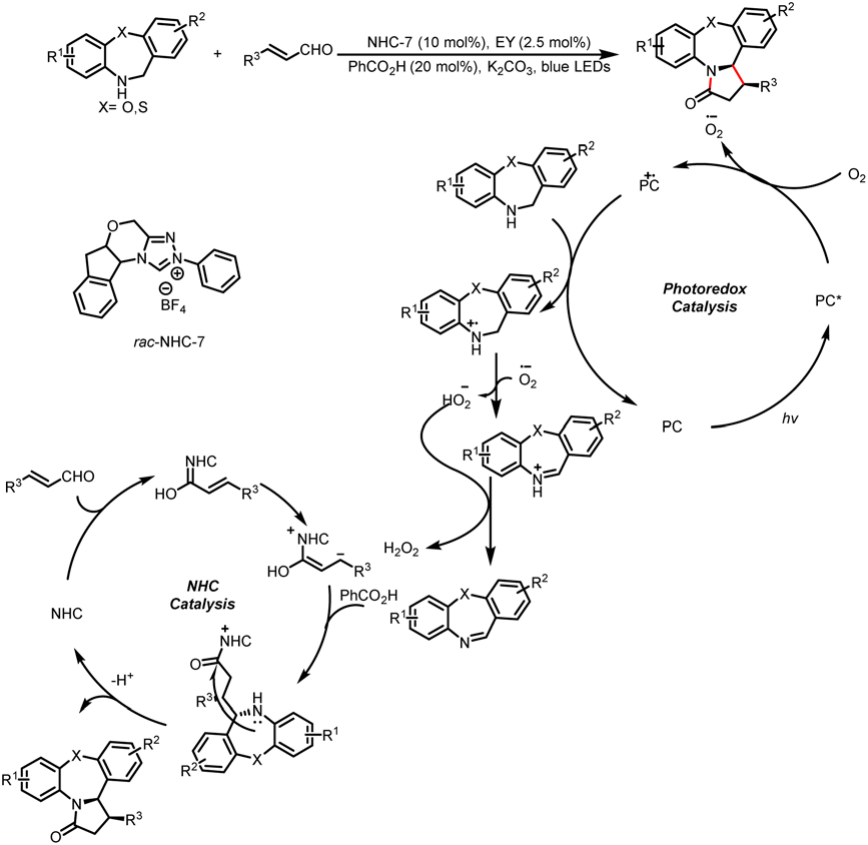
Synthesis of pyrrolo[1,2-*d*][1,4]oxazepin-3(2*H*)-ones.

Recently, the Chauhan group developed a stereoselective strategy to access pyrrolo[1,2-*d*][1,4]-oxazepin-3(2*H*)-ones (Scheme 16).^21^ Imine is formed upon one-electron oxidation, hydrogen atom transfer, and proton transfer of amine. Then the imine intermediate enters into the NHC catalytic cycle, where the Breslow intermediate acts as the homoenolate equivalent, which after tautomerization undergoes nucleophilic addition to imine to forge acyl azolium species. Eventually the azolium intermediate delivers the product.

### 2.2 Reactions not requiring an external photocatalyst

**Scheme 17:**
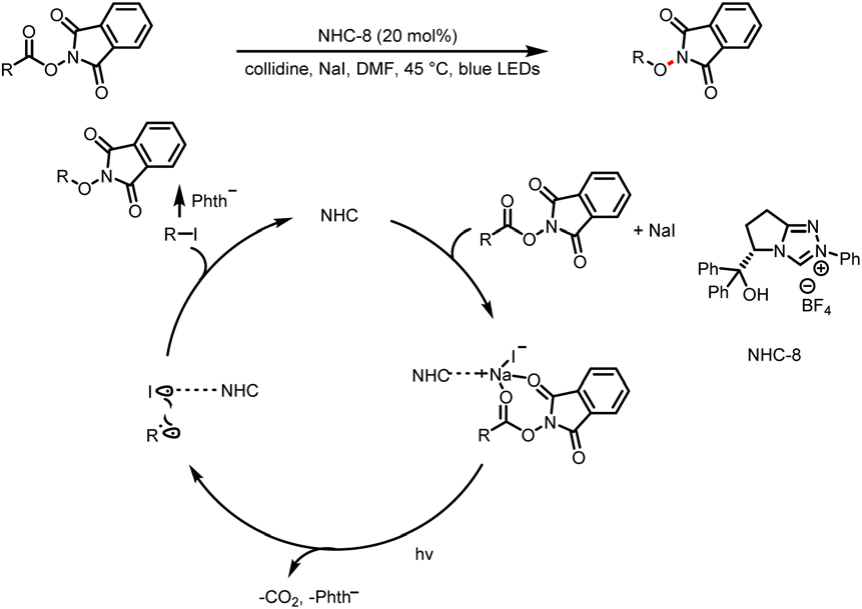
Synthesis of protected amines.

**Scheme 18:**
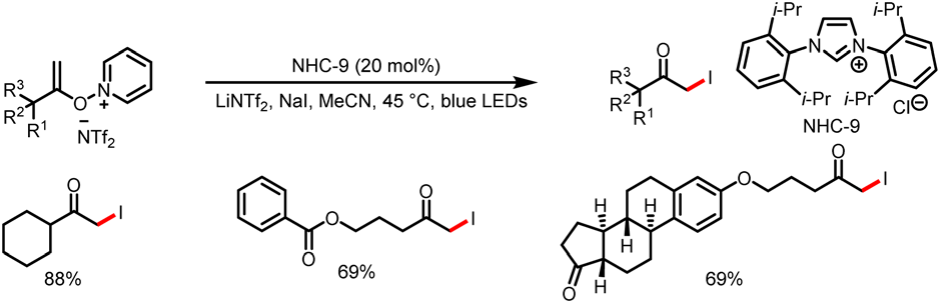
Synthesis of α-iodo ketones.

The process without an external photocatalyst is usually to reduce the radical precursor by generating an excited state of the Breslow intermediate under light excitation, directly generate free radicals through special radical precursors under light or generate EDA complexes through the Breslow intermediates and other reactants in the system. Various reactions can be achieved by combining light activation and NHC organocatalysis without an external photocatalyst. For example, in 2020, Chen's group demonstrated that transition-metal-free decarboxylative C(sp^3^)–X bond formation can be accomplished with photochemically active *N*-(acyloxy)phthalimide ester–NaI–NHC complexes; these reactions offer a convenient way to construct C(sp^3^)–C(sp^2^), C(sp^3^)–S, C(sp^3^)–O, and C(sp^3^)–Cl bonds (Scheme 17).^22^ The key to these reactions is the electrostatic NHC–Na^+^ interaction, which facilitates the formation of electron donor–acceptor complexes, irradiation of which generates radicals that go on to form the products. Later, the same research group described catalytic reactions of *N*-alkenoxypyridinium salts and NaI to give various α-iodo ketones (Scheme 18).^23^

**Scheme 19:**
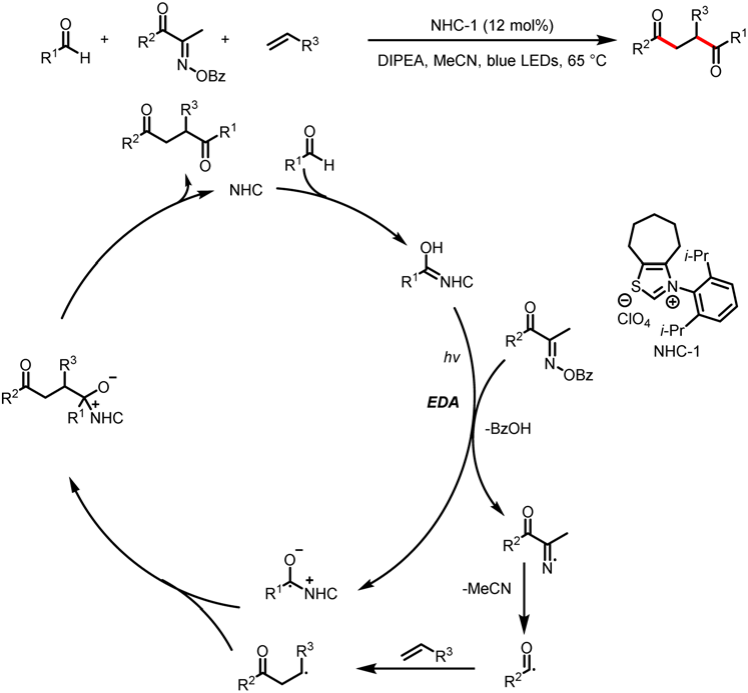
Synthesis of 1,4-diketones from oximes, aldehydes, and alkenes.

In 2022, the Larionov group developed a method for visible-light-induced NHC-catalyzed regioselective 1,2-diacylation reactions of alkenes that afford 1,4-diketones *via* three-component C–C-bond-forming radical coupling (Scheme 19).^24^ Notably, in this system, the NHC catalyst plays two roles: an NHC-catalyst-derived intermediate forms an electron donor–acceptor complex, and the NHC catalyst acts as an acyl transfer reagent. Under irradiation by blue LEDs, intramolecular SET between a Breslow intermediate and an oxime generates nitrogen and ketyl radicals. The nitrogen radical fragments to release acetonitrile and an acyl radical, which adds to an olefin to generate an adduct radical. The adduct radical participates in a cross-coupling reaction with the ketyl radical to afford a 1,4-diketone in high yield.

**Scheme 20:**
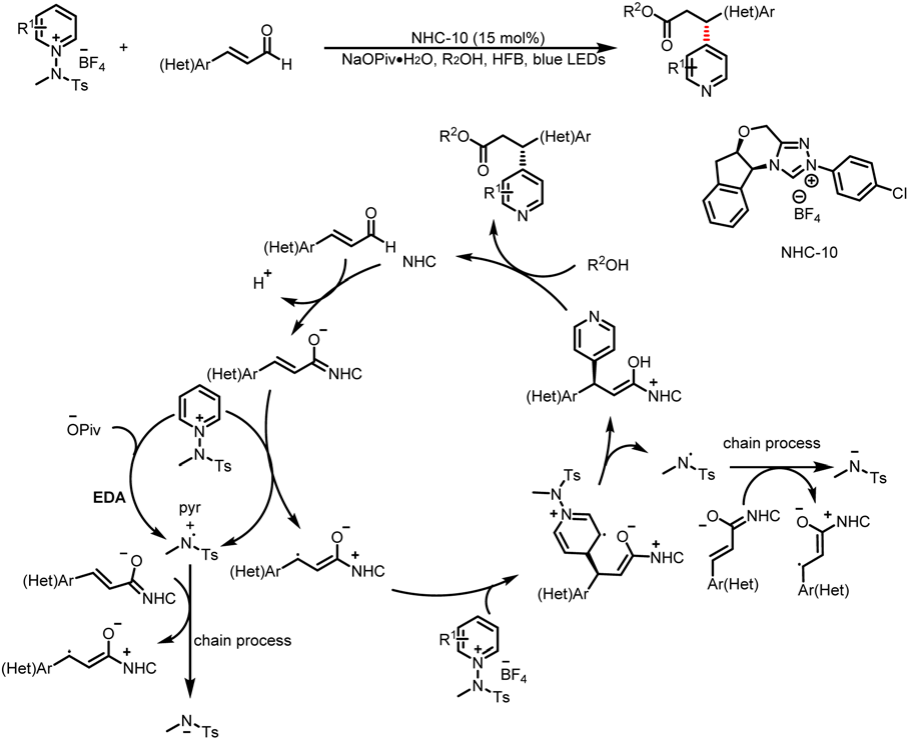
Synthesis of β-pyridylated esters.

The Hong group developed a catalytic method for enantioselective, C4-selective functionalization of pyridine derivatives (Scheme 20),^25^ which proceeds by means of a mechanism similar to that reported by Larionov *et al.*^24^ The key to these asymmetric β-pyridylation reactions of enals is an enhanced interactions between a chiral–NHC–bound homoenolate and a pyridinium salt in the presence of a pivalate salt and hexafluorobenzene, which effectively distinguishes between the two faces of a homoenolate radical. Because light-facilitated reactivity and rate acceleration were observed, Hong *et al.*^25^ proposed an alternative mechanism involving photon absorption by a pyridine–pivalate electron donor–acceptor complex that triggers the formation of amidine radicals when the EDA complex is irradiated with visible light.

**Scheme 21:**
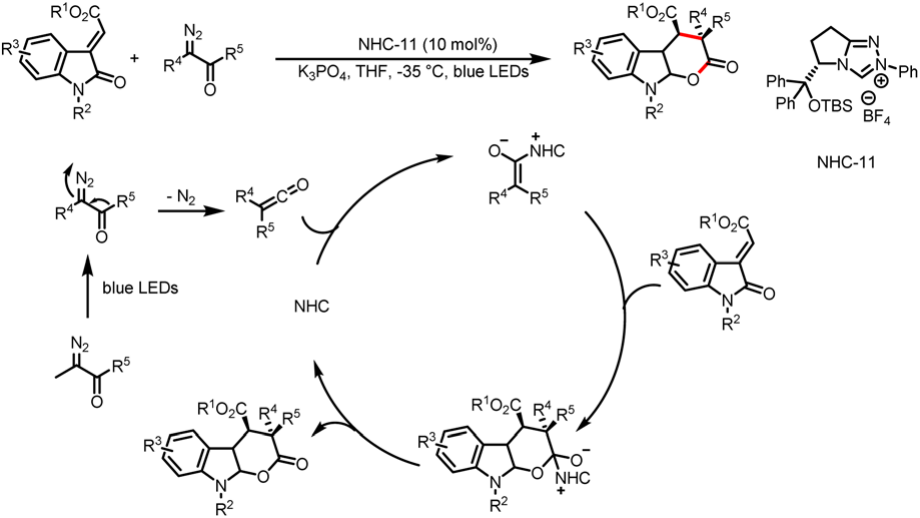
Synthesis of tetrahydropyrano[2,3-*b*]indoles.

**Scheme 22:**
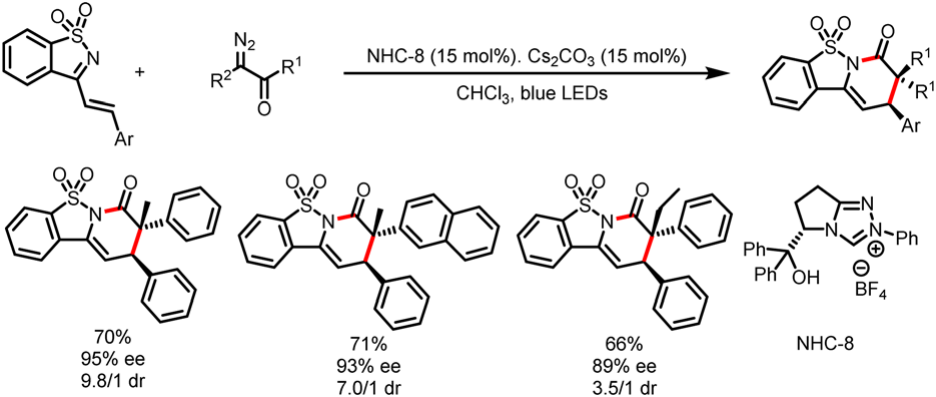
Synthesis of dihydropyridinones.

Other systems can, upon photoirradiation, directly generate radicals that can then participate in a catalytic cycle involving NHC. In 2020, the Hui group achieved stereoselective [4 + 2] cycloaddition reactions of 3-alkylenyloxindoles and α-diazoketones that proceed *via* this mechanism (Scheme 21).^26^ Initially, a ketene is formed from the α-diazoketone through a Wolff rearrangement reaction under blue light. Subsequently, the addition of NHC to the ketene generates an enolate, which participates in a [4 + 2] annulation reaction with the 3-alkylenyloxindole to give an intermediate that is transformed into a tetrahydropyrano[2,3-*b*]indole upon release of the NHC catalyst. Subsequently, the Yao group developed a method for asymmetric [4 + 2] annulation reactions of saccharine-derived azadienes and α-diazoketones, affording the corresponding sultam-fused dihydropyridinones in moderate to good yields with satisfactory to excellent enantio- and diastereo-selectivities (Scheme 22).^27^

**Scheme 23:**
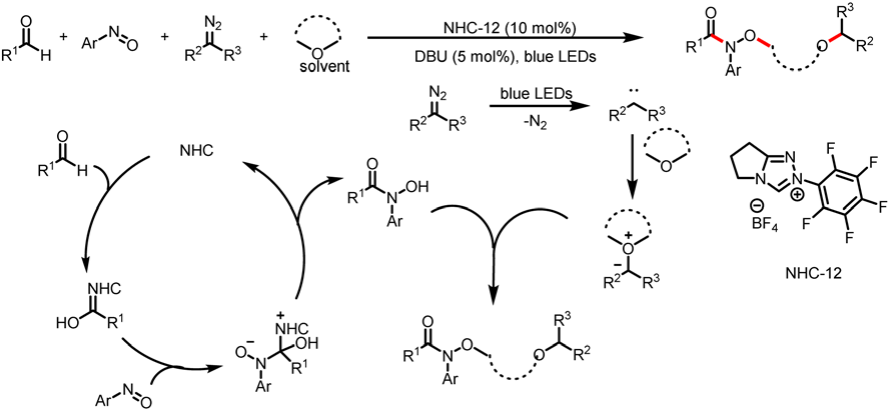
Formation of hydroxamic acid esters by a four-component reaction in a cyclic ether as a reaction medium.

In 2020, the Xuan group described a multicomponent reaction that relies on the different reactivities of two carbenes (Scheme 23).^28^ One of the N-heterocyclic carbenes, acting as an organocatalyst, mediates the formation of a hydroxamic acid *in situ*, whereas the other carbene, which is formed by photolysis of diazoalkane, acts as a reactant. Then the hydroxamic acid and carbene participate in a solvent-dependent four-component reaction that provides hydroxamic acid esters, which are biologically important compounds.

## 3. Ketyl radicals generated *via* acyl azolium intermediates

### 3.1 Acyl imidazoles as acyl sources

#### 3.1.1 Direct formation of benzyl radicals

**Scheme 24:**
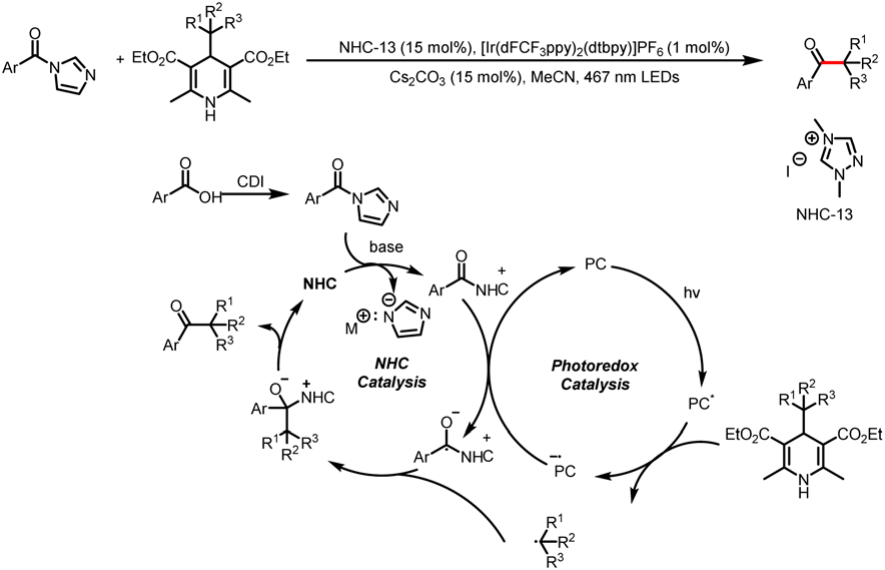
Synthesis of ketones from HEs and aryl acyl imidazoles.

Like processes involving single-electron oxidation of electron-rich Breslow intermediates, processes involving generation of electron-deficient acyl azolium intermediates and their single-electron reduction are currently a booming area of research. In 2020, Scheidt *et al.* reported a method for reductive single-electron alkylation of acyl azoliums to form ketones from aryl acyl imidazoles derived from carboxylic acids (Scheme 24).^29^ Hantzsch esters (HEs), which can be easily synthesized from simple starting materials with a variety of structures, are used as alkylation reagents. The excited-state photocatalyst initially oxidizes the HE to an alkyl radical (most of the examples involve a benzyl radical), and single-electron reduction of the acyl azolium ion provides a ketyl radical while regenerating the ground-state photocatalyst. Radical–radical coupling and loss of the NHC afford the desired ketone. Note, however, that this reaction is applicable only to aryl carboxylic acids.

**Scheme 25:**
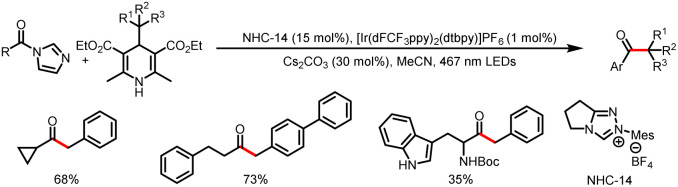
Synthesis of ketones from HEs and alkyl acyl imidazoles.

Scheidt *et al.* later expanded the reaction to alkyl acyl imidazoles by altering the structure of the NHC catalyst. Their findings revealed that both electronic and steric modifications of the NHC catalyst would affect the stability and accessibility of the radical intermediates, thereby controlling the reactivity of NHC catalysis (Scheme 25).^30^

**Scheme 26:**
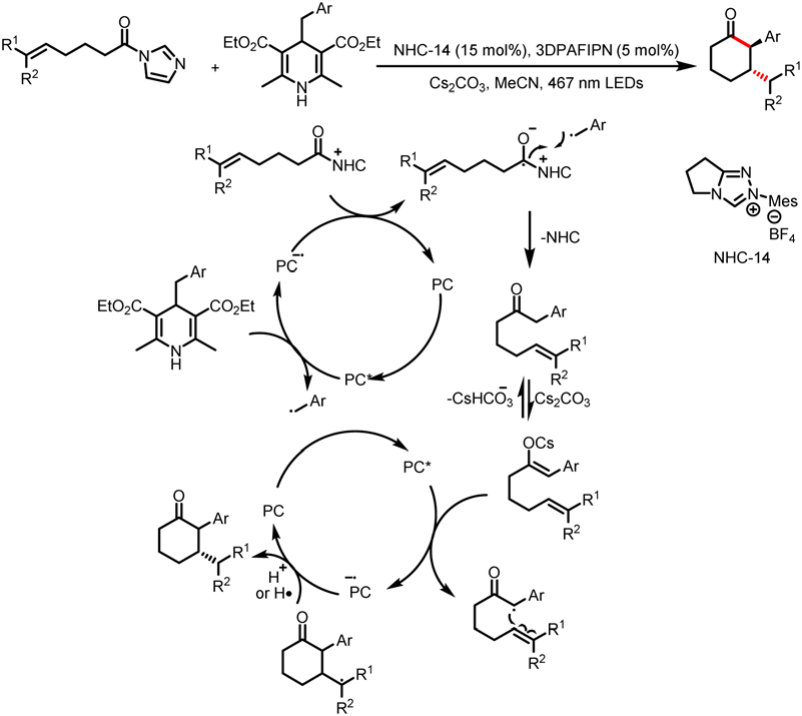
Synthesis of α,β-disubstituted cyclohexanones from HEs and alkyl acyl imidazoles.

In 2022, the Scheidt group developed a strategy for the construction of two contiguous C–C bonds *via* a formal [5 + 1] cycloaddition for the synthesis of α,β-disubstituted cyclohexanones from HEs and alkyl acyl imidazoles (Scheme 26).^31^ The key to this transformation lies in the two photocatalytic cycles to achieve intramolecular cyclization. In the first cycle, the photoexcited photocatalyst oxidizes the HE to afford a benzyl radical, and single-electron reduction of an acyl azolium provides a ketyl radical. Intermolecular coupling of the two radicals and loss of the NHC give a linear ketone intermediate. In the second cycle, the corresponding enol or cesium enolate undergoes single-electron oxidation to produce a benzyl radical, which participates in an intramolecular cyclization reaction with the remote double bond. The resulting radical undergoes hydrogen atom transfer (HAT) or is reduced to the corresponding anion by the photocatalyst, and subsequent deprotonation by the solvent or bicarbonate affords the desired cyclohexanone product.

**Scheme 27:**
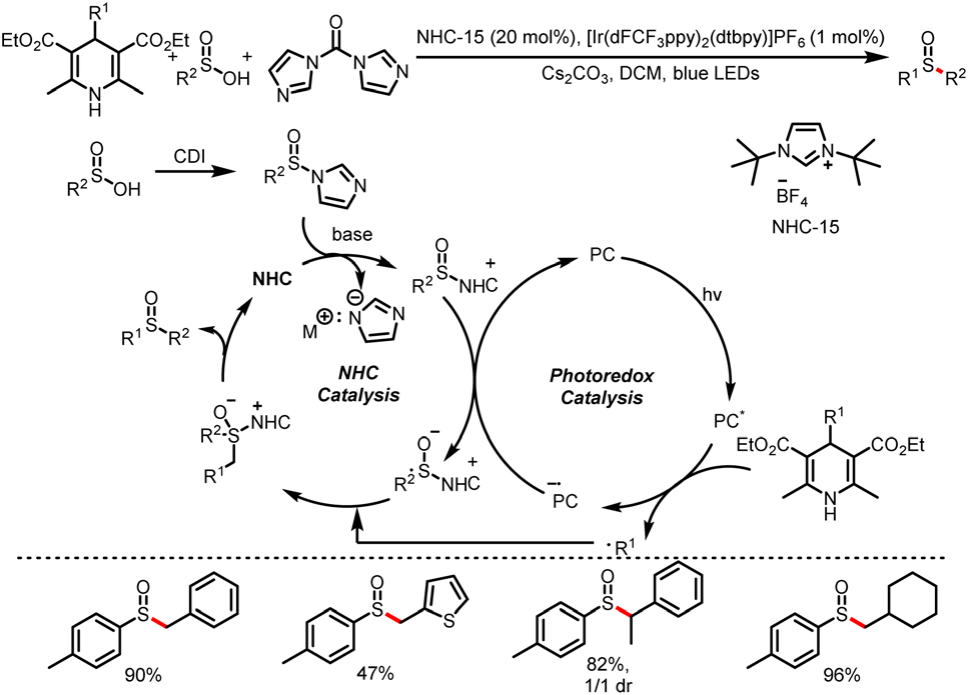
Synthesis of sulfoxides from HEs and sulfinyl imidazoles.

Subsequently, the Wu group reported the generation of sulfoxides from sulfinic acids and 4-substituted HEs in the presence of carbonyldiimidazole (Scheme 27).^32^ Acting as free-radical precursors, HEs can generate not only benzyl radicals but also alkyl radicals, and a series of sulfinyl products were obtained by this method. This method further expands the previous carboxylic acid category to sulfonic acid, and using sulfinyl imidazoles as pseudo-acyl sources.

#### 3.1.2 Indirect formation of benzyl radicals

**Scheme 28:**
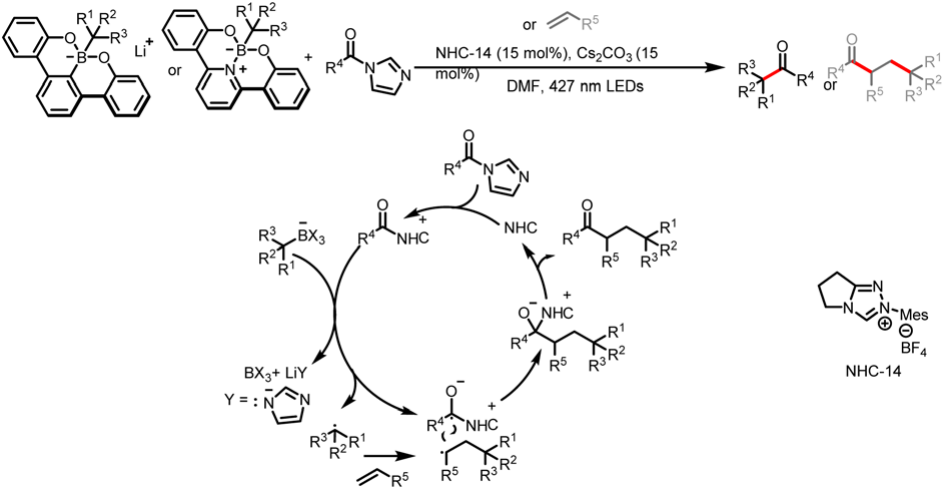
Synthesis of ketones from alkylborates and alkyl acyl imidazoles.

In addition to being directly accessible by single-electron oxidation of radical precursors, benzyl radicals can also be obtained indirectly by the addition of radicals to styrene analogues. In 2021, Ohmiya and co-workers designed a protocol that enables cross-coupling between alkylborates and alkyl acyl imidazoles in addition to radical relay-type alkylacylations of alkenes with alkylborates and alkyl acyl imidazoles, affording a diverse array of ketones (Scheme 28).^33^ In a light-driven NHC catalytic cycle, an alkyl radical and a ketyl radical, generated from the borate or the acyl azolium intermediate by a SET process, participate in radical–radical coupling to yield the acylation product. Alternatively, the addition of the alkene to the reaction system results in a radical relay process, and subsequent radical–radical coupling between another alkyl radical and the ketyl radical affords the alkylacylation product.

**Scheme 29:**
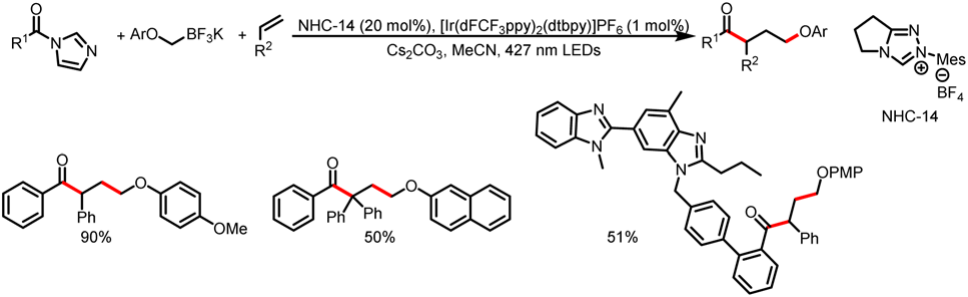
Synthesis of γ-aryloxyketones.

In 2022, the Scheidt group reported a multicomponent reaction for the synthesis of γ-aryloxyketones *via* aryloxymethyl potassium trifluoroborate salts. An aryloxymethyl radical adds to a styrene derivative to provide a stabilized benzyl radical, and a subsequent radical–radical coupling reaction with an azolium radical affords the γ-aryloxyketone product (Scheme 29).^34^

**Scheme 30:**
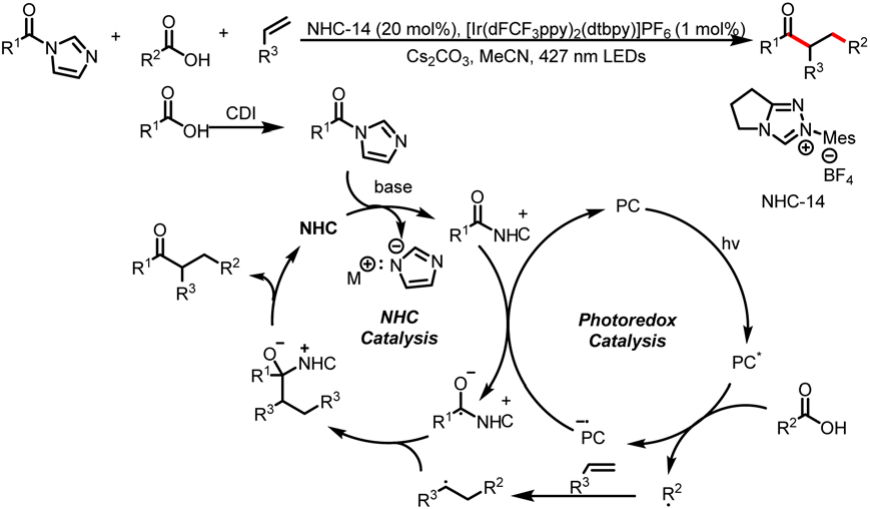
Synthesis of ketones from carboxylic acids and acyl imidazoles.

In the same year, the Chi group published a method for coupling carboxylic acids and acyl imidazoles by means of a combination of NHC catalysis and photocatalysis (Scheme 30).^35^ The carboxylic acids are directly used as radical precursors, and late-stage modification of commercial drugs and direct coupling of fragments of two medicinally active molecules were performed to demonstrate the utility of this method.

**Scheme 31:**
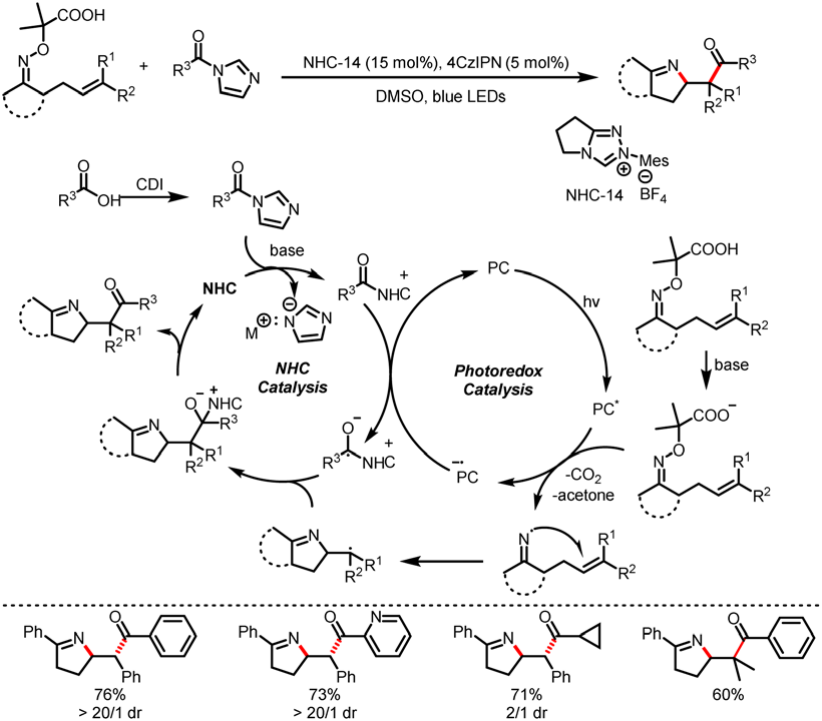
Synthesis of substituted 3,4-dihydro-2*H*-pyrroles from oxime ethers and acyl imidazoles.

Recently, the Ye group reported the iminoacylation of alkenes *via* decarboxylation of α-imino-oxy acids to generate iminyl radical intermediates. The addition of the iminyl radicals to a tethered alkene in a 5-*exo-trig* manner gives dihydropyrrole-derived carbon radicals (most of the examples were benzyl radicals), which couple with ketyl radicals generated from acyl azolium intermediates to form substituted 3,4-dihydro-2*H*-pyrroles (Scheme 31).^36^

#### 3.1.3 Formation of heteroatomic ortho radicals

**Scheme 32:**
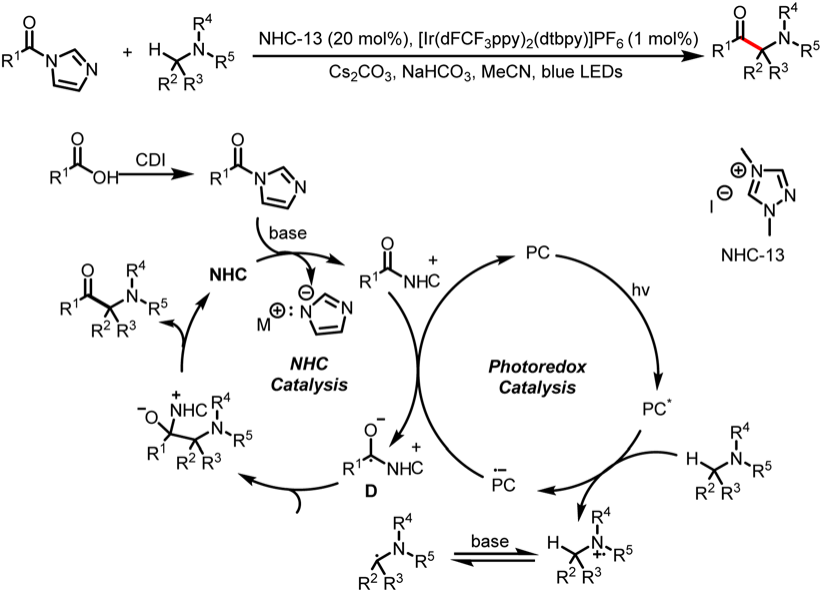
Construction of α-amino ketones from acyl imidazoles and amines.

In 2022, the Wang group reported the direct acylation of α-C(sp^3^)–H bonds of amines by acyl imidazoles to access α-amino ketones (Scheme 32).^37^ Single-electron oxidation of the amine and subsequent deprotonation afford an α-amino radical, and then radical–radical cross-coupling with a ketyl radical provides the corresponding product.

#### 3.1.4 Formation of allyl radicals

**Scheme 33:**
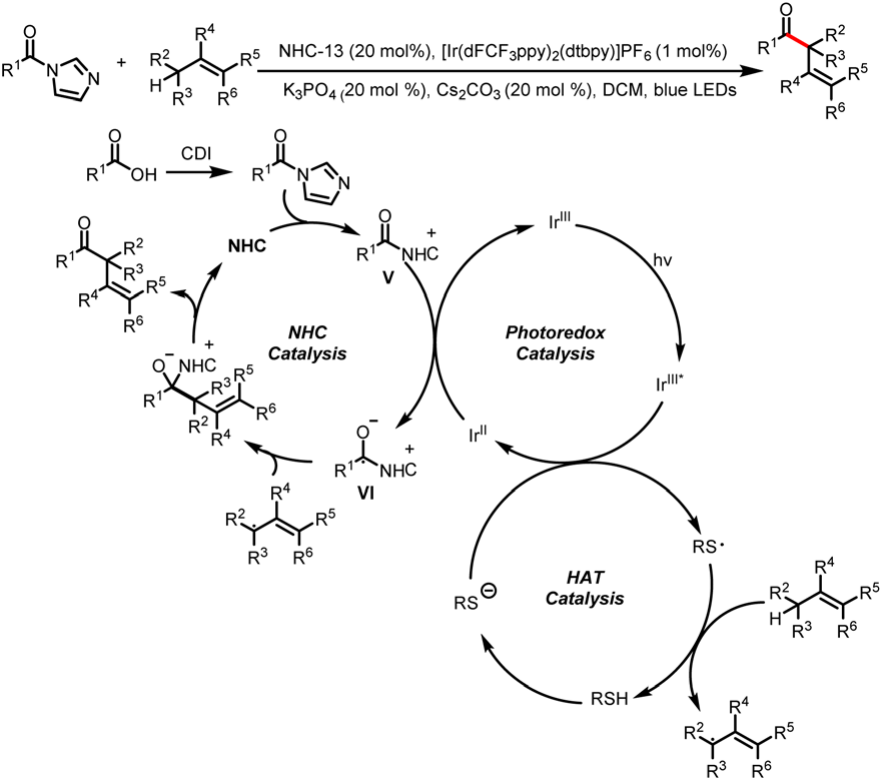
Construction of β,γ-unsaturated ketones from acyl imidazoles and alkenes.

Recently, the Wang group achieved the direct allylic acylation *via* cross-coupling involving cooperative NHC, hydrogen atom transfer (HAT), and photoredox catalysis to synthesize β,γ-unsaturated ketones (Scheme 33).^38^ The thiyl radical generated from single-electron oxidation of thiol serves as a powerful HAT catalyst, abstracting an allylic hydrogen from alkenes to generate allyl radicals. The coupling of allyl radicals with ketyl radicals affords desired β,γ-unsaturated ketones.

### 3.2 Acyl fluorides as acyl sources

#### 3.2.1 Direct formation of benzyl radicals

**Scheme 34:**
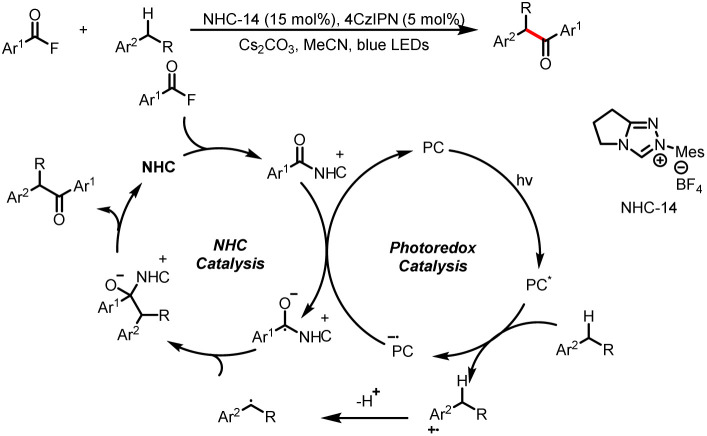
Construction of benzyl aryl ketones by benzylic C(sp^3^)-aroylation.

In addition to imidazole, fluorine can also be used as a leaving group in single-electron reduction of acyl azolium intermediates to generate ketyl radicals. For example, in 2021, the Studer group developed a method for direct acylation of benzylic C–H bonds *via* NHC/photoredox dual catalysis (Scheme 34).^39^ This mild method allows the preparation of a range of benzylic ketones and shows a good functional group. Under irradiation of blue LEDs, single-electron oxidation of electron-rich alkylarenes and subsequent deprotonation at the benzylic position give the corresponding benzyl radicals, which couple with ketyl radicals derived from acyl azolium intermediates to afford benzyl aryl ketones.

**Scheme 35:**
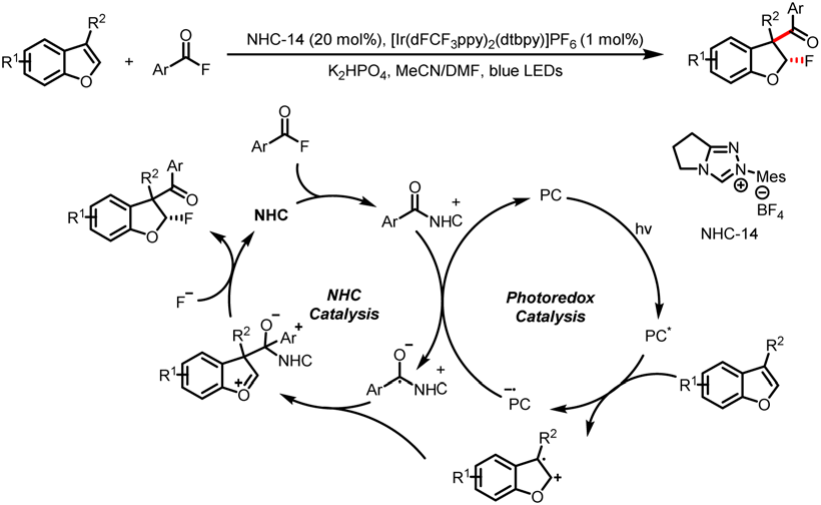
Construction of 3-aroyl-2-fluoro-2,3-dihydrobenzofurans from acyl fluorides and benzofurans.

In addition, this research group also generated benzyl radicals by means of SET oxidation of the double bonds of benzofurans (Scheme 35).^40^ Specifically, they reported fluoroaroylation of benzofurans by acyl fluorides, which act as bifunctional reagents to incorporate both an aroyl moiety and fluorine into the product. Upon visible-light irradiation, the benzofuran is oxidized to a radical cation by a photoexcited photocatalyst. A ketyl radical is generated from an acyl azolium intermediate, and cross-coupling of the radical cation and the ketyl radical leads to an oxocarbenium ion. Diastereoselective trapping of this ion by a F anion affords the 3-aroyl-2-fluoro-2,3-dihydrobenzofuran product.

**Scheme 36:**
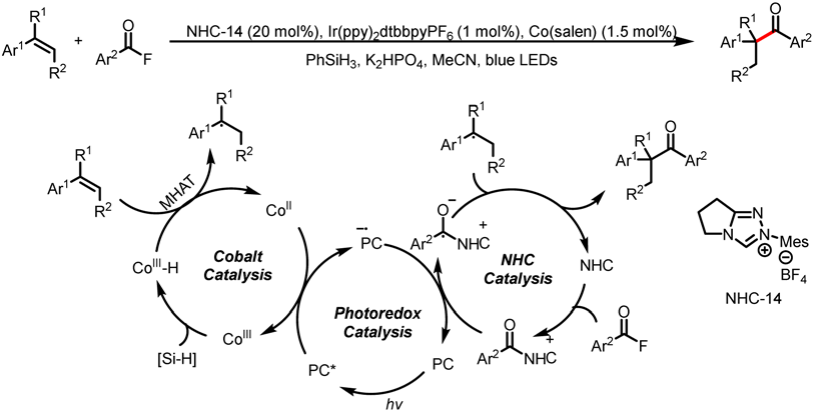
Markovnikov-selective hydroacylation of alkenes.

Like benzofurans, substituted styrenes can act as benzyl radical precursors by undergoing metal-mediated HAT. In 2022, the Wang group achieved Markovnikov-selective hydroacylation of alkenes by using a synergistic combination of cobalt, photoredox, and NHC catalysis (Scheme 36).^41^ The cobalt catalytic cycle starts with SET oxidation of Co^II^ to Co^III^, and then Co^III^ is captured by phenylsilane to furnish a Co^III^–H intermediate. This intermediate engages in a HAT reaction with the substituted styrene to produce a benzyl radical. Meanwhile, SET reduction of an acyl azolium ion gives a ketyl radical, which undergoes radical–radical cross-coupling with the benzyl radical to generate the hydroacylation product.

**Scheme 37:**
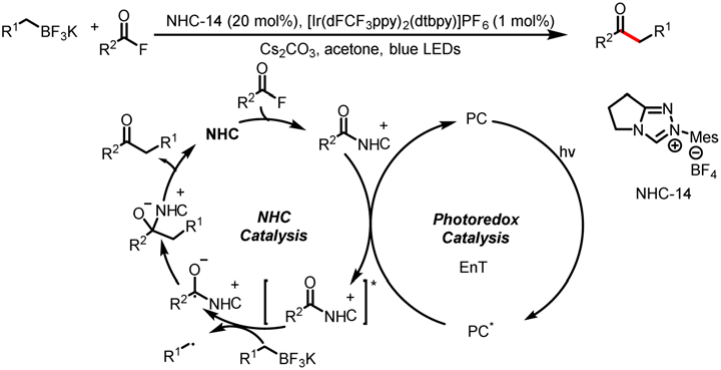
Construction of ketones from acyl fluorides and alkyl trifluoroborates.

Shortly thereafter, the Li group reported cross-coupling reactions of alkyl trifluoroborates with acid fluorides to generate various ketones; this method provides an alternative to the classical acylative Suzuki coupling chemistry (Scheme 37). Li *et al.* proposed the activation of an acyl azolium intermediate with a triplet-state photocatalyst, through an energy-transfer process, to form an excited-state acyl azolium intermediate.^42^ This intermediate oxidizes the alkyl trifluoroborates to give a ketyl radical and a benzyl radical. The coupling of these two radicals affords the ketone product.

#### 3.2.2 Indirect formation of benzyl radicals

**Scheme 38:**
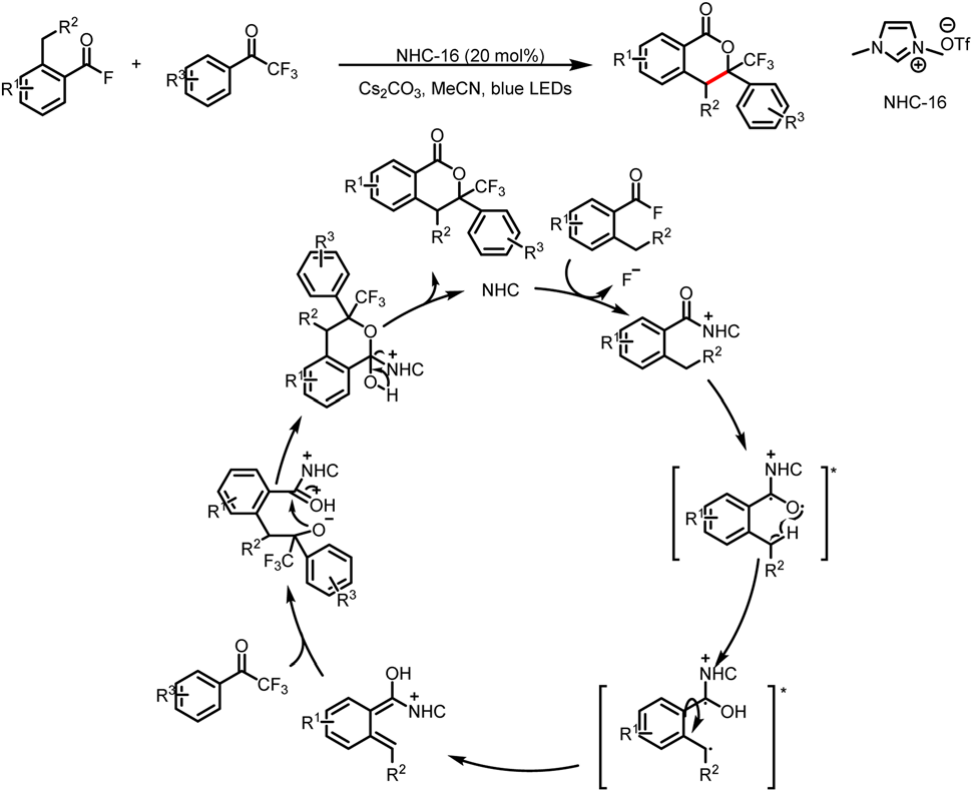
Synthesis of isochroman-1-one derivatives.

In 2020, the Hopkinson group reported the use of acid fluorides and trifluoroacetophenones as substrates in a UVA-light-mediated photochemical transformation that leads to diverse isochroman-1-one derivatives (Scheme 38).^43^ The azolium intermediate generated from the acid fluoride by NHC catalysis is excited by UVA irradiation to afford, after intersystem crossing, a triplet excited state. Then 1,5-HAT from the *o*-benzylic position to the radical-like carbonyl oxygen atom gives rise to a triplet-state dienol biradical. Rotation of this species before relaxation leads to a ground-state intermediate, which can react with the dienophile in a cycloaddition process. Finally, elimination of the NHC from the cycloadduct completes the catalytic cycle.

**Scheme 39:**
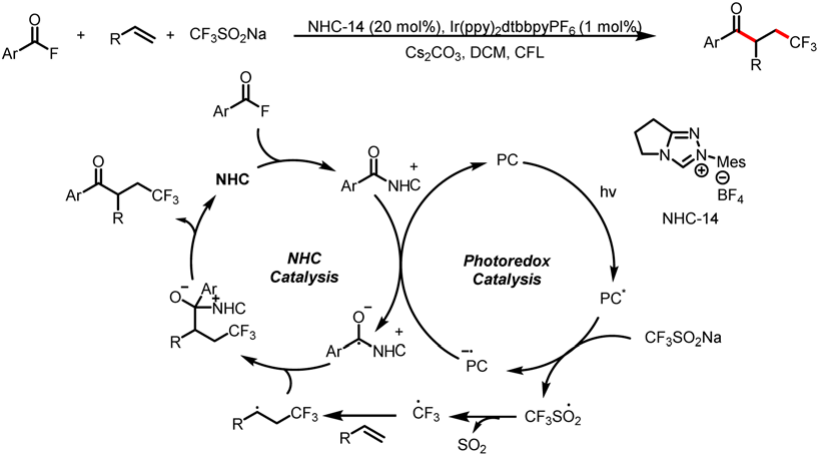
Construction of β-trifluoromethyl-α-substituted ketones from acyl fluorides and styrenes.

In 2020, the Studer group reported three-component coupling reactions of aroyl fluorides, styrenes, and the Langlois reagent (CF_3_SO_2_Na) to give various β-trifluoromethylated-α-substituted ketones (Scheme 39).^44^ Reductive quenching of the excited-state photocatalyst by the trifluoromethanesulfinate anion gives a trifluoromethylsulfonyl radical that fragments to release SO_2_ and a trifluoromethyl radical, which then adds to the double bond of styrene to generate a transient benzylic radical. Meanwhile, SET reduction of an acyl azolium ion gives a ketyl radical, which undergoes radical–radical cross-coupling with the benzyl radical to give the β-trifluoromethyl-α-substituted ketones.

**Scheme 40:**
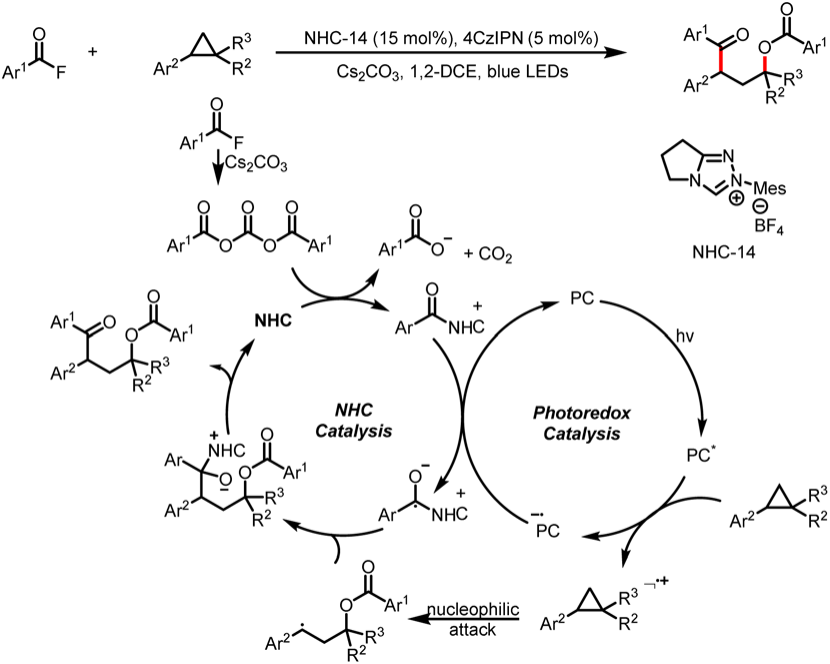
Construction of γ-aroyloxy ketones from acyl fluorides and aryl cyclopropanes.

Shortly thereafter, Studer *et al.* reported a ring-opening/arylcarboxylation/acylation cascade reaction for the 1,3-difunctionalization of aryl cyclopropanes (Scheme 40).^45^ The key to this transformation is that the aryl cyclopropane radical cation generated by SET oxidation of the aryl cyclopropane undergoes ring opening by a nucleophilic benzoate ion to give a benzylic radical. Radical–radical cross-coupling of the benzylic radical and a ketyl radical affords γ-aroyloxy ketones.

**Scheme 41:**
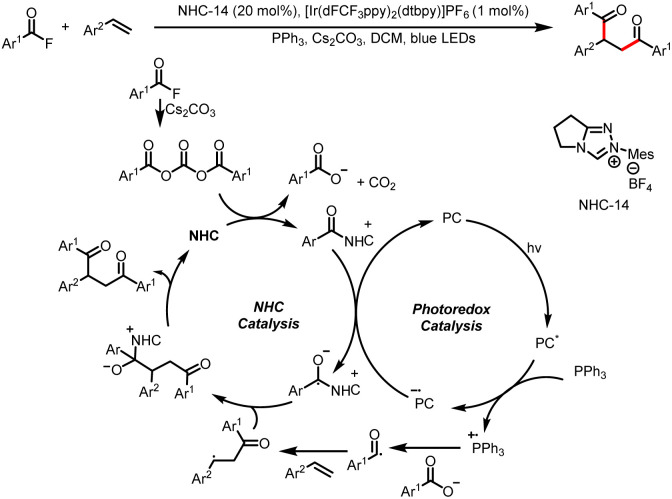
Construction of symmetrical 1,4-dicarbonyl compounds from acyl fluorides and styrenes.

**Scheme 42:**
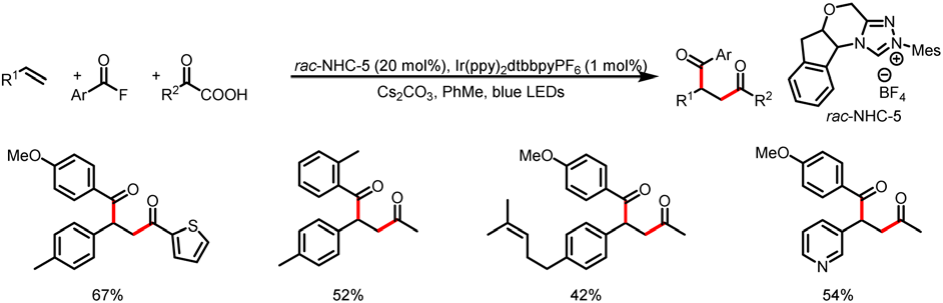
Construction of unsymmetrical 1,4-dicarbonyl compounds from alkenes, acyl fluorides, and keto acids.

The Feng and Fan group developed a method for intermolecular 1,2-diacylation of styrenes *via* cooperative NHC and photoredox catalysis with mediation by PPh_3_ and Cs_2_CO_3_ (Scheme 41).^46^ The mechanism is similar to that described by Studer *et al.*^45^ An NHC-mediated reaction of a bisacyl carbonate intermediate generated from an acyl fluoride produces a benzoate anion and an acyl azolium ion. The benzoate anion combines with a triphenylphosphine cation radical to form a phosphorus-centered radical, which undergoes β-scission to generate an acyl radical. The acyl radical attacks the styrene substrate to generate the corresponding benzyl radical. Next, intermolecular cross-coupling between a ketyl radical (derived from the acyl azolium ion) and the benzyl radical produces the desired product. In a similar manner, keto acids can also be used as acyl radical precursors *via* single-electron oxidation, and subsequent dicarbonylation of alkenes provides direct access to 1,4-dicarbonyl compounds (Scheme 42).^47^

**Scheme 43:**
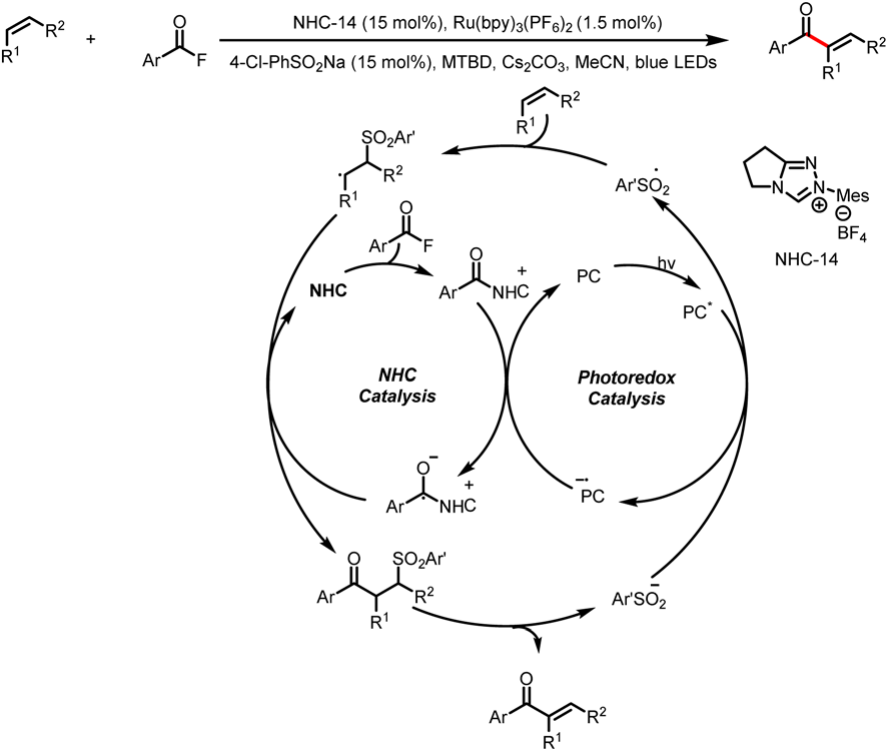
Construction of α-substituted vinyl ketones by α-acylation of alkenes.

Unlike trifluoromethylsulfonyl radicals, aryl sulfonyl radicals do not undergo SO_2_ extrusion to produce the corresponding aryl radicals. Therefore, in 2022, Studer and a colleague developed an arylsulfonate-catalyzed alkene acylation reaction. In this reaction, an arylsulfonyl radical first adds to the alkene substrate to generate a carbon-centered radical, which then couples with a ketyl radical (generated from an acyl azolium), providing a three-component coupling intermediate. The subsequent base-mediated elimination of arylsulfinate forms the α-acylated olefin product. Three catalytic cycles involving a carbene are interwoven (Scheme 43).^48^

**Scheme 44:**
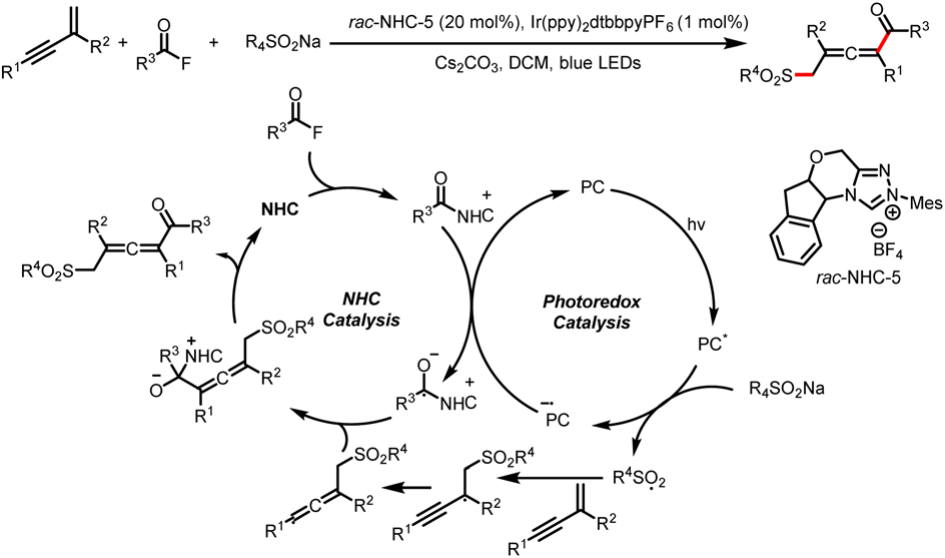
1,4-Sulfonylacylation of 1,3-enynes.

**Scheme 45:**
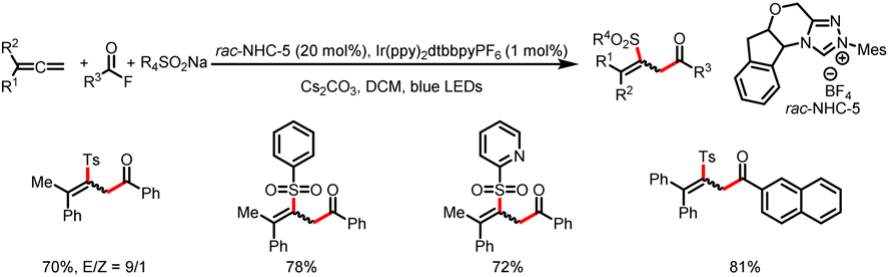
1,2-Sulfonylacylation of allenes.

The Zhang group described a similar reaction: 1,4-sulfonylacylation of 1,3-enynes to provide valuable structurally diverse tetrasubstituted allenyl ketones (Scheme 44).^49^ The addition of a sulfonyl radical to a 1,3-enyne delivers a propargyl radical that can undergo reversible isomerization to generate a trisubstituted allenyl radical, which can participate in a radical–radical cross-coupling reaction with a ketyl radical generated by reduction of an acyl azolium ion. By replacing the 1,3-enyene acceptor of the sulfonyl radical with an allene, these investigators also achieved 1,2-sulfonylacylation of allenes to provide valuable sulfonyl-containing multisubstituted allyl ketones (Scheme 45).^50^

#### 3.2.3 Formation of phenyl radicals

**Scheme 46:**
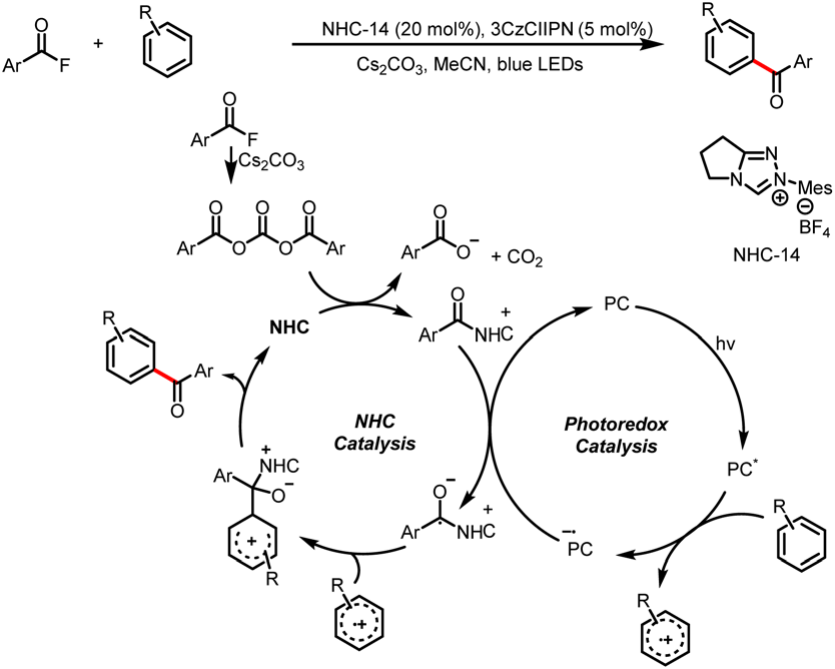
C–H Acylation of arenes.

Recently, the Studer group reported a C–H Acylation of arenes and heteroarenes through cooperative photoredox/NHC radical catalysis (Scheme 46).^51^ The cross coupling of arene radical cations, formed through the single electron oxidation of arenes, with an NHC-bound ketyl radical leads to ketone products. Importantly, if acylation occurs under classical Friedel–Crafts conditions using AlCl_3_ as a Lewis acid, different regioselectivity will be obtained. Therefore, by simply switching the reaction conditions, two different regional isomers can be obtained.

#### 3.2.4 Formation of carbamoyl radicals

**Scheme 47:**
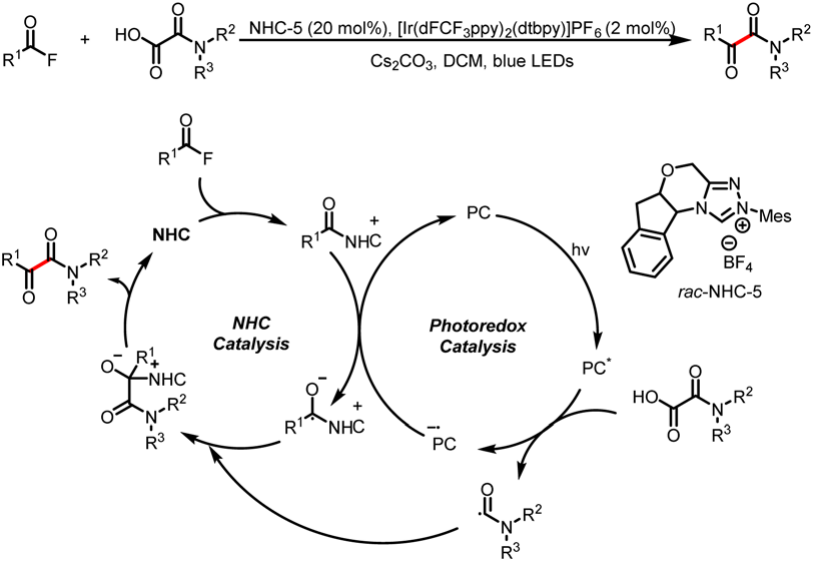
Construction of α-amino ketones from acyl fluoride and oxamic acid.

Recently, the Luo and Yang group reported a decarboxylativeacylation of oxamic acid with acyl fluoride to produce α-keto amides (Scheme 47).^52^ A carbamoyl radical is generated through single-electron oxidation and then couples with a ketyl radical to produce the desired product.

### 3.3 Esters as acyl sources

**Scheme 48:**
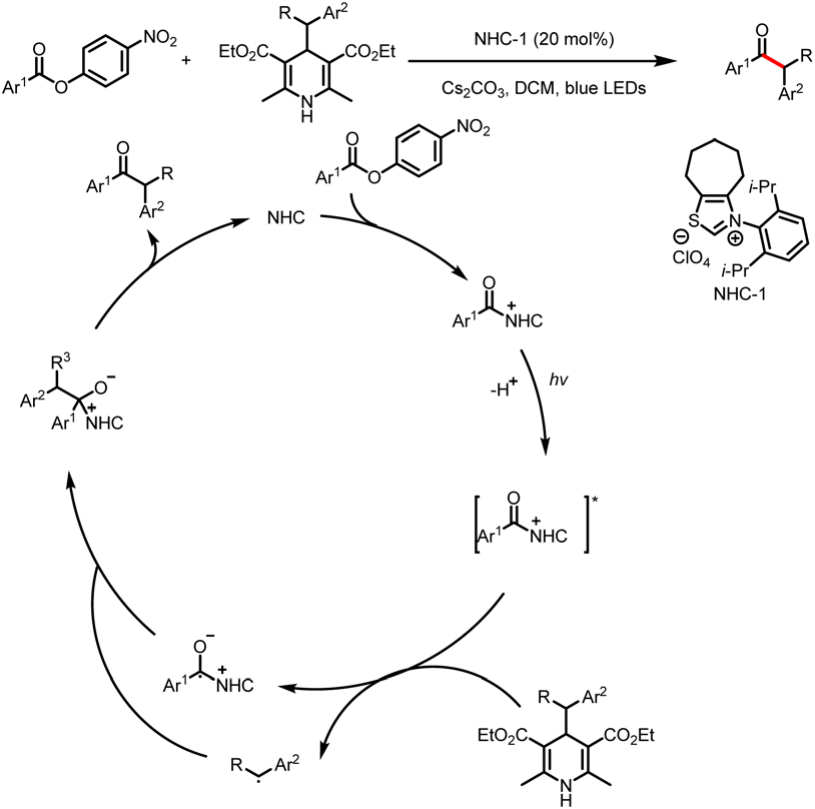
Alkylation of carboxylic esters to generate ketones.

In addition to imidazoles and fluorides, esters can also act as leaving groups. For example, in 2021, the Chi group developed a method for alkylation of aryl carboxylic esters with HEs (Scheme 48).^53^ The reaction starts with addition of an NHC catalyst to the ester to generate an acyl azolium intermediate. The photoexcitation of this intermediate converts it to an excited state that act as a single-electron oxidant. Subsequently, SET between the HE and the excited-state acyl azolium leads to a benzyl radical and a ketyl radical, and coupling of these two radicals affords the desired ketone product. Structurally sophisticated ketones, including ketones bearing medicinal fragments, could readily be prepared.

## 4. Conclusion and outlook

As complements to two-electron reaction modes, single-electron reaction modes give new vitality to NHC-catalyzed reactions. This review has focused on combinations of NHC catalysis with photocatalysis. The combinations have been classified on the basis of whether the ketyl radical is generated by single-electron oxidation of an electron-rich Breslow intermediate or by single-electron reduction of an electron-deficient acyl azolium intermediate. Reactions in the first category have been further categorized on the basis of their substrates and whether an external photocatalyst is required, whereas reactions in the second category have been categorized mainly on the basis of the type of leaving group and the type of radical that is generated. Through the combination of NHC catalysis and photocatalysis, many transformations that cannot be achieved by means of two-electron reactions can be realized.

Although NHC-mediated single-electron reactions have made great progress and breakthroughs, there are still some problems that remain to be solved, including: (1) acyl substrate scope: since the previously activated acyl precursors are mostly carboxylic acids, it would be important to explore whether the activation process of carboxylic acid derivatives, aldehyde derivatives and imines could be realized through the co-catalysis mode of light and NHC; (2) types of free radicals: since most of the literature reported was on the direct formation of benzyl radicals or the addition of alkyl radicals to aryl olefins to achieve the indirect formation of benzyl radicals, to explore other unstable radical such as selective and efficient acylation of unactivated C–H bonds through this catalytic mode will be meaningful; (3) enantioselectivity control: how to build a quaternary or tertiary stereocenter at the α-position of a carbonyl group with high enantioselectivity should be considered; (4) catalytic mode: the development of new catalytic modes through merging NHC catalysis with otherwise established chemistry protocols such as electrochemistry or metal catalysis should be explored as well.

## Author contributions

Xiaochen Wang: conceptualization, visualization, and writing – original draft. Senhui Wu and Rongxin Yang: Investigation. Hongjian Song and Yuxiu Liu: writing – review & editing. Qingmin Wang: supervision, conceptualization, writing – review & editing, and funding acquisition. All authors have given approval to the final version of the manuscript.

## Conflicts of interest

There are no conflicts to declare.

## Supplementary Material
